# Investigating the effect of national government physical distancing measures on depression and anxiety during the COVID-19 pandemic through meta-analysis and meta-regression

**DOI:** 10.1017/S0033291721000933

**Published:** 2021-03-02

**Authors:** João M. Castaldelli-Maia, Megan E. Marziali, Ziyin Lu, Silvia S. Martins

**Affiliations:** Department of Epidemiology, Mailman School of Public Health, Columbia University, New York, NY 10032, USA

**Keywords:** Anxiety, COVID-19, depression, public transport, social isolation

## Abstract

**Background:**

COVID-19 physical distancing measures can potentially increase the likelihood of mental disorders. It is unknown whether these measures are associated with depression and anxiety.

**Objectives:**

To investigate meta-analytic global levels of depression and anxiety during the COVID-19 pandemic and how the implementation of mitigation strategies (i.e. public transportation closures, stay-at-home orders, etc.) impacted such disorders.

**Data sources:**

PubMed, MEDLINE, Web of Science, BIOSIS Citation Index, Current Content Connect, PsycINFO, CINAHL, medRxiv, and PsyArXiv databases for depression and anxiety prevalences; Oxford Covid-19 Government Response Tracker for the containment and closure policies indexes; Global Burden of Disease Study for previous levels of depression and anxiety.

**Study eligibility criteria:**

Original studies conducted during COVID-19 pandemic, which assessed categorical depression and anxiety, using PHQ-9 and GAD-7 scales (cutoff ⩾10).

**Participants and interventions:**

General population, healthcare providers, students, and patients. National physical distancing measures.

**Study appraisal and synthesis methods:**

Meta-analysis and meta-regression.

**Results:**

In total, 226 638 individuals were assessed within the 60 included studies. Global prevalence of both depression and anxiety during the COVID-19 pandemic was 24.0% and 21.3%, respectively. There were differences in the prevalence of both anxiety and depression reported across regions and countries. Asia (17.6% and 17.9%), and China (16.2% and 15.5%) especially, had the lowest prevalence of both disorders. Regarding the impact of mitigation strategies on mental health, only public transportation closures increased the prevalence of anxiety, especially in Europe.

**Limitations:**

Country-level data on physical distancing measures and previous anxiety/depression may not necessarily reflect local (i.e. city-specific) contexts.

**Conclusions and implications of key findings:**

Mental health concerns should not be viewed only as a delayed consequence of the COVID-19 pandemic, but also as a concurrent epidemic. Our data provide support for policy-makers to consider real-time enhanced mental health services, and increase initiatives to foster positive mental health outcomes.

## Introduction

COVID-19 is an unprecedented health emergency, affecting millions of individuals across the globe (Velavan & Meyer, 2020). SARS-Coronavirus-2, the virus which causes COVID-19, is transmitted person-to-person via respiratory droplets (Wiersinga, Rhodes, Cheng, Peacock, & Prescott, 2020). In order to prevent and lessen spread, countries began implementing mitigation strategies, such as stay-at-home or shelter-in-place orders, international travel constraints, closure of schools and workplaces, and movement limitations (Hale et al., 2020). Despite taking necessary public health measures, researchers have speculated that these measures could increase feelings of social isolation and loneliness (Marziali et al., 2020); this is of importance, as previous studies have demonstrated that social isolation could impact the likelihood of mental disorders (Torales, O'Higgins, Castaldelli-Maia, & Ventriglio, 2020) and physical health outcomes (House, Landis, & Umberson, 1988). As of yet, it still remains unclear to what extent the COVID-19 mitigation strategies could impact mental health. Thus, it is imperative to investigate the levels of mental health disorders and the possible impacts of social distancing measures on mental health outcomes (Carvalho Aguiar Melo & de Sousa Soares, 2020).

Before the pandemic, depression and anxiety were the most prevalent mental health disorders in the world (GBD 2017 Diseases and Injuries Collaborators et al., 2018). Depression can affect one in every five people in some countries (Bromet et al., 2011); anxiety disorders could be even more prevalent, with more than a quarter of individuals reporting these disorders during the lifetime in some countries (Kessler et al., 2007). These mental health disorders have also been connected to social isolation during COVID-19 in local studies (Al-Qahtani, Elgzar, & Ibrahim, 2020). During the COVID-19 pandemic, the levels of such disorders have increased. A meta-analysis with 13 studies that included 33 062 healthcare workers during COVID-19 reported a prevalence of 23.2% and 22.8% for anxiety and depression, respectively (Pappa et al., 2020). These prevalences are greater than those found in the pre-COVID-19 era (GBD 2017 Diseases and Injuries Collaborators, 2018). Several studies have assessed depression and anxiety using scales involving self-reporting during the pandemic (Ahmad, Rahman, & Agarwal, 2020; Ahn et al., 2020; Ahorsu et al., 2020; Alyami et al., 2020; Amerio et al., 2020; Bachilo, Barylnik, Shuldyakov, Efremov, & Novikov, 2020; Bauer et al., 2020; Bauerle et al., 2020; Chang, Yuan, & Wang, 2020; Chen et al., 2020; Choi, Hui, & Wan, 2020; Civantos et al., 2020; Consolo, Bellini, Bencivenni, Iani, & Checchi, 2020; Fancourt, Steptoe, & Bu, 2020; Filho et al., 2020; Gao et al., 2020; Guo et al., 2020; Hu et al., 2020; Islam, Ferdous, & Potenza, 2020; Jia et al., 2020; Johnson, Ebrahimi, & Hoffart, 2020; Juanjuan et al., 2020; Kantor & Kantor, 2020; Khanna, Honavar, Metla, Bhattacharya, & Maulik, 2020; Killgore, Cloonan, Taylor, & Dailey, 2020; Lai et al., 2020; Lin et al., 2020; Liu, Zhang, Wong, Hyun, & Hahm, 2020a; J. Liu et al., 2020b; Mahendran, Patel, & Sproat, 2020; Mechili et al., 2020; Muñoz-Navarro, Vindel, Schmitz, Cabello, & Fernández-Berrocal, 2020; Naser et al., 2020; Nguyen et al., 2020; Olaseni, Akinsola, Agberotimi, & Oguntayo, 2020; Pieh, Budimir, & Probst, 2020; Qian et al., 2020; Que et al., 2020; Saddik, Hussein, Albanna, et al., 2020a; Saddik, Hussein, Sharif-Askari, et al., 2020b; Salman, Asif, et al., 2020a; Salman, Raza, et al., 2020b; Shi et al., 2020; Sigdel et al., 2020; Solomou & Constantinidou, 2020; Stickley, Matsubayashi, Sueki, & Ueda, 2020; Stojanov et al., 2020; Sun, Goldberg, Lin, Qiao, & Operario, 2020; Tang et al., 2020; Temsah et al., 2020; Ueda, Stickley, Sueki, & Matsubayashi, 2020; Wang et al., 2020; Weilenmann et al., 2020; Xiao et al., 2020; Yamamoto, Uchiumi, Suzuki, Yoshimoto, & Murillo-Rodriguez, 2020; Zhang et al., 2020; Zhao, Peng, Liu, & Ouyang, 2020a; R. Zhao et al., 2020b; Zhou et al., 2020; Zhu et al., 2020), and the geographic location within which the study is focused. There is a need for meta-analytic investigations generating global prevalence measures for both depression and anxiety during the pandemic, with additional exploration via subgroup analysis.

Further, there are mixed findings regarding the effect of mitigation strategies on depression and anxiety during this pandemic. Previous research has demonstrated marked increases in online search trends for mental health topics (i.e. sleep disturbances, negative thoughts, anxiety, suicidal ideation) prior to the implementation of stay-at-home orders in the USA (Jacobsen et al., 2020). Further, an online qualitative study evaluated focus groups during the beginning of the social distancing measures in the UK, where they found negative impacts on well-being and mental health after implementation of mitigation strategies (Williams, Armitage, Tampe, & Dienes, 2020). Individuals who had lower pay, or vulnerable employment, were the most affected (Williams et al., 2020). Thus, the effects of these physical distancing strategies may be time-sensitive. Moreover, there are varying ongoing physical distancing measures (i.e. school closures, workplace closures, public events cancellations, restrictions on the size of gatherings, public transport closures, stay-at-home orders, restrictions on internal movement between cities and regions within a country, and international travel controls) during different periods, depending on the location (Hale et al., 2020). There is a need to explore whether these strategies have lasting impacts on depression and anxiety, taking different time of exposure thresholds to such physical distancing measures into account.

The present study aims to (1) investigate meta-analytic global levels of depression and anxiety during the COVID-19 pandemic, and (2) explore the effects of these mitigation strategies on depression and anxiety.

## Methods

### Study design

We first conducted a meta-analysis of studies related to the COVID-19 pandemic which assessed depression and anxiety using PHQ-9 and GAD-7 scales. Subgroup analysis for region of the world, country, type of population, and coverage was also carried out. Then, we collected national data regarding the implementation of physical distancing measures and mitigation strategies (Hale et al., 2020), along with the previous levels of anxiety and depression from a global database (GBD 2017 Diseases and Injuries Collaborators et al., 2018). These data were included in meta-regression models for the investigation of time-sensitive effects of mitigation strategies on depression and anxiety, adjusted for previous levels of such disorders and other possible confounders.

### Review guidelines and registration

This study followed the PRISMA statement for a transparent report of systematic reviews and meta-analysis (Moher, Liberati, Tetzlaff, Altman, & Group, 2009) and MOOSE guidelines for Meta-analysis Of Observational Studies in Epidemiology (Stroup et al., 2000). Online Supplementary Figs S1 and S2, respectively, present PRISMA and MOOSE checklists reporting the page of the manuscript in which we consider that each item was addressed. This study was registered at the Center for Open Science/Open Science Framework (Castaldelli-Maia, 2020).

### Search strategy

We searched PubMed, MEDLINE, Web of Science, BIOSIS Citation Index, Current Content Connect, PsycINFO, and CINAHL databases. All searches were conducted with an end date of 29 July 2020. Search terms used were: [(sars-cov-2 OR coronavir* OR alphacoronavirus OR betacoronavirus OR COVID OR COVID-19) AND (PHQ-9 or GAD-7)]. As this topic is developing quickly, we accessed pre-print servers medRxiv and PsyArXiv using the above search terms. We also searched the WHO database which includes COVID literature (cite) for studies published by the same date, using the following search terms: (PHQ-9 or GAD-7). In addition to MEDLINE, this database also includes WHO COVID, Elsevier, Lanzhou University/CNKI, LILACS, and WPRIM databases.

### Screening and eligibility

**Fig. 1.**
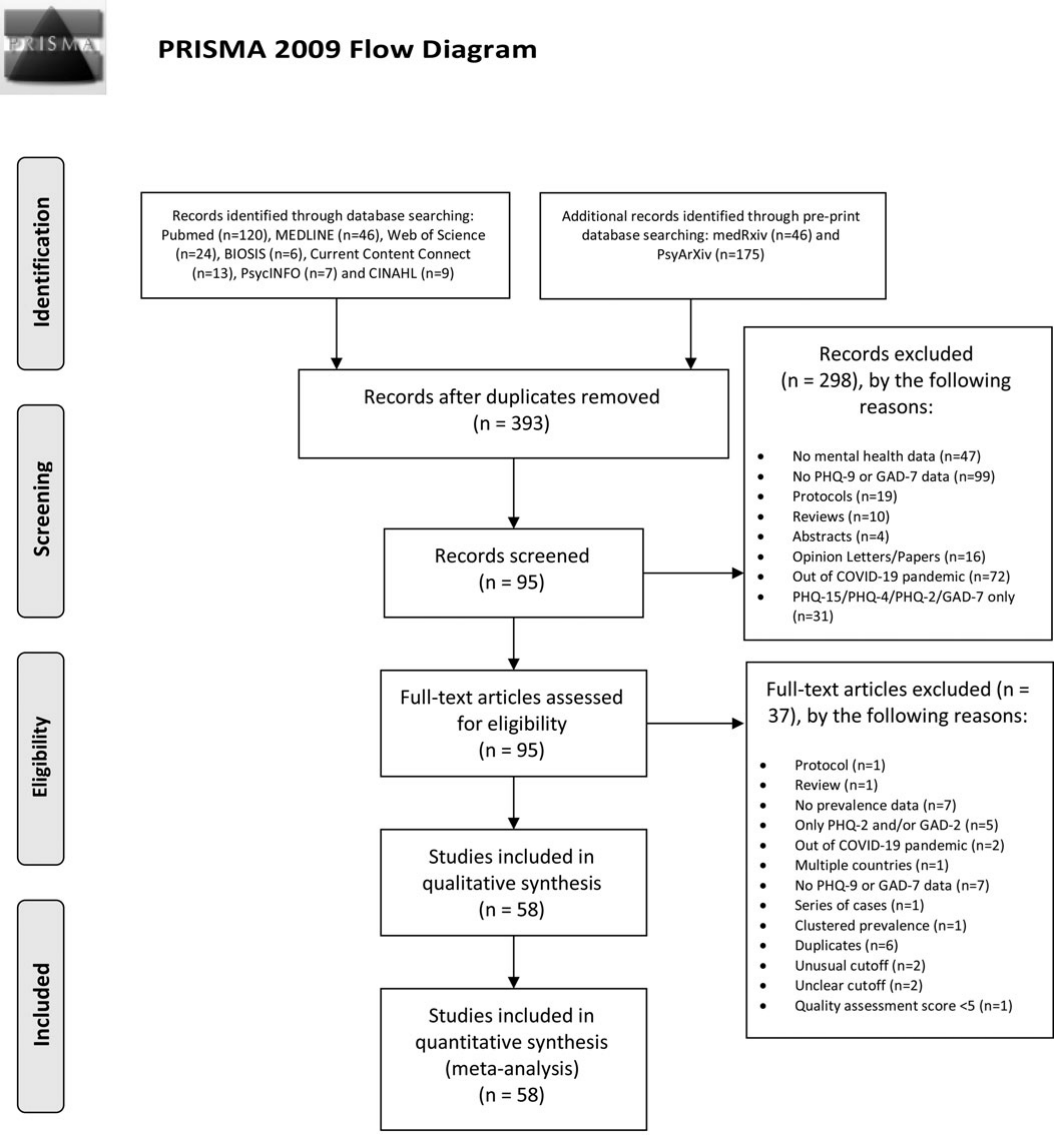
PRISMA Flow Diagram.

We first removed duplicates from our search results. Screening and eligibility were performed by three researchers independently (JMCM, MEM, ZL). Studies that were written in Chinese were screened by two researchers (JMCM, ZL). Disagreement on the inclusion of a study based on the title or abstract resulted in the study being retained for the next screening stage. We did not find articles in languages other than English and Chinese. Reasons for the exclusion of full texts were collected and presented in the PRISMA Flow Diagram (Fig. 1).

We included studies that reported categorical assessment of anxiety and depression using GAD-7 and PHQ-9 scales during the COVID-19 pandemic. Randomized controlled trials, cohort studies, case–control studies, and cross-sectional studies were included. Pre-prints and letters were included if they described the original research.

### Data extraction

Data were extracted by two of the three independent reviewers (JMCM, MEM, ZL). Descriptive variables extracted were setting (i.e. country), population type (e.g. pregnant women and children), study design (e.g. cohort and case–control), follow-up time, nature of the control group, number of cases, number of controls, age, and gender. Randomized controlled trials, for this review, were treated as cohort studies. The timepoint for data extraction in prospective studies was either before the intervention (i.e. clinical trials) or during the COVID-19 pandemic (i.e. cohort studies). Data were stored in Excel version 16.16.11.

### Quality assessment

The purpose of this appraisal was to assess the methodological quality of the included studies and to determine the extent to which a study has addressed the possibility of bias in its design, conduct and analysis. All studies included in the present systematic review were subjected to the Joanna Briggs Institute Checklist for Analytical Cross-Sectional Studies (The Joanna Briggs Institute, 2017), which assesses sample frame, process and size, setting description, data analysis coverage, valid and reliable assessment methods, appropriate statistical analysis, and an adequate response rate.

### Measures

Apart from outcome (depression and anxiety) and exposure (physical distancing measures) variables that are further explained, the present study sought the following data from each included study: the number of individuals enrolled in the study; mean age, standard deviation, and minimum/maximum age range of participants (or median and interquartile range); the proportion of women included; whether the study was nationally representative; whether the study was peer-reviewed; format of data collection (i.e. online); and geographic location, including city, state, and country. Subsequently, we collected data on the prevalence of depression and anxiety, prior to the COVID-19 pandemic, within each country included a review from the Global Burden of Disease (GBD) Study 2017; this data source reports estimated prevalence and burden due to anxiety and depression for all the countries included in the present study. Data contained within the GBD were extracted from censuses, household surveys, civil registration and vital statistics, disease registries, health service use, air pollution monitors, satellite imaging, disease notifications, and other sources (GBD 2017 Diseases and Injuries Collaborators et al., 2018).

#### Anxiety and depression

The Patient Health Questionnaire-9 (PHQ-9) (Kroenke, Spitzer, & Williams, 2001) is a screening instrument for depressive disorders. It is composed of nine basic items based on the DSM-IV diagnostic criteria for major depressive disorder. The questions assess the frequency of depressive symptoms in the last 2 weeks. The respondents answer on a scale from 0 (not at all) to 3 (nearly every day). Several studies have used the cut-off ⩾10 to define clinically relevant depression (online Supplementary Table S1). The Generalized Anxiety Disorder-7 (GAD-7) is a screening instrument for anxiety symptoms (Spitzer, Kroenke, Williams, & Löwe, 2006). The GAD-7 is a validated scale that measures anxiety with seven self-rating items on a four-point scale, similarly to PHQ-9. A cut-off ⩾10 has been used by several studies to define clinically relevant anxiety (online Supplementary Table S1). Both the PHQ-9 and GAD-7 have excellent psychometric properties (Kroenke et al., 2001; Spitzer et al., 2006).

#### Exposure: implementation of physical distancing strategies

We collected national data from the Oxford Covid-19 Government Response Tracker (Hale et al., 2020). All containment and closure policies were included in the present study, as follows:

- School closures (0 – no measures; 1 – recommend closing; 2 – require closing only some levels or categories; 3 – require closing all levels);
- Workplace closures (0 – no measures; 1 – recommend closing or recommend work from home; 2 – require closing or work from home for some sectors or categories of workers; 3 – require closing or work from home for all-but essential workplaces);
- Cancellation of public events (0 – no measures; 1 – recommend cancelling; 2 – require cancelling);
- Restrictions on gatherings (0 – no restrictions; 1 – restrictions on very large gatherings above 1000 people; 2 – restrictions on gatherings between 101 and 1000 people; 3 – restrictions on gatherings between 11 and 100 people; 4 – restrictions on gatherings of 10 people or less);
- Public transportation closures (0 – no measures; 1 – recommend closing or significantly reduce volume/route/means of transport available; 2 – require closing or prohibit most citizens from using it);
- Stay at home requirements (0 – no measures; 1 – recommend not leaving house; 2 – require not leaving house with exceptions for daily exercise, grocery shopping, and ‘essential’ trips; 3 – require not leaving house with minimal exceptions);
- Restrictions on internal movement: record restrictions on internal movement between cities/regions (0 – no measures; 1 – recommend not to travel between regions/cities; 2 – internal movement restrictions in place); and
- International travel controls: record restrictions on international travel for foreign travelers (0 – no restrictions; 1 – screening arrivals; 2 – quarantine arrivals from some or all regions; 3 – ban arrivals from some regions; 4 – ban on all regions or total border closure).

For each study included in the meta-analysis, we calculated the mean of the daily ordinal score of each of the above indexes, during two timeframes:

- 2-week: weeks before the start date of the study until the end date of the study; and
- 4-week: weeks before the start date of the study until the end date of the study.

### Statistical analysis

We included all the rates (crude number of cases/total number of individuals) in separate meta-analysis models for depression (PHQ-9 ⩾ 10) and anxiety (GAD-7 ⩾ 10). One study provided weighted rates for the outcomes only (Fancourt, Steptoe, & Bu, 2020). We used a random-effects model because high heterogeneity was expected. We calculated *I*^2^ as a measure of between-study heterogeneity. Data were analyzed using OpenMetanalyst (Wallace et al., 2012), which makes use of R metafor package (Viechtbauer, 2010). The threshold for significance was set to *p* values of less than 0.05. In addition, we carried out further subgroup analysis models by population type (general, healthcare providers, students, patients, and mixed), region of the world (Asia, Europe, and Other), country (China and other), income level (high-income, and low- and middle-income), and non-national status (local studies were defined as those restricted to either a city or a state/province/region within a country, *v.* national studies).

Finally, we investigated the impact of physical distancing measures on depression and anxiety through meta-regression models (Higgins & Thompson, 2004). Separate models were carried out for different timeframes of physical distancing measures (2 and 4 weeks). Models adjusted for gender, sub-populations, timepoint when study began, region of the world, local status, and previous levels of either depression or anxiety, depending on the outcome. Country indicators were not included in these models because of the strong correlation with earlier levels of depression and anxiety variables, which were collected based on previous data by each country. Meta-regression was used instead of subgroup analyses (i.e. different levels of social measures implementation) to allow for the use of continuous and multiple covariates. The random-effects meta-regression used residual restricted maximum likelihood to measure between-study variance (*τ*2) with a Knapp-Hartung modification as recommended models (Higgins & Thompson, 2004). In the case of significant results for physical distancing measures, sensitivity analyses were carried out including adjustments for peer-review status and quality assessment scores of the studies.

## Results

Online Supplementary Table S1 presents the key-information of the 60 studies included. Eight studies were split into subsamples, and two studies reported the same sample. We included 67 samples in the meta-analysis models. All studies were conducted in 2020 (compiled date range of study initiation to closure: 24 January–31 May), with a mean length of 15.4 days. In total, 226 638 individuals were included, with an average of 3382 individuals per study. The mean age was 33.8 (range: 13–89) among samples that provided data on mean age and range, and the proportion of females included was 61.9% (range: 0–100). Few samples were representative (5.9%, *N* = 4), and local (32.8%, *N* = 22). Most samples were based in China (38.8%, *N* = 26), and Asia in general (52.2%, *N* = 35). General population samples were the most common (40.2%, *N* = 27), followed by healthcare providers (23.8%, *N* = 16), students (16.4%, *N* = 11), and patients (8.9%, *N* = 6). The vast majority of the samples used online methods (91.0%, *N* = 61) and were peer-reviewed (64.1%, *N* = 43). Online Supplementary Table S2 presents the results of the quality assessment. All the included studies scored five or higher in such an assessment. Online Supplementary Tables S3 and S4 present implementation of physical distancing measures and previous prevalence of depression and anxiety, respectively. Differences in prevalences were found across all countries. Depression and anxiety prevalences were 2.4% and 4.2% on average (online Supplementary Table S4).

**Fig. 2.**
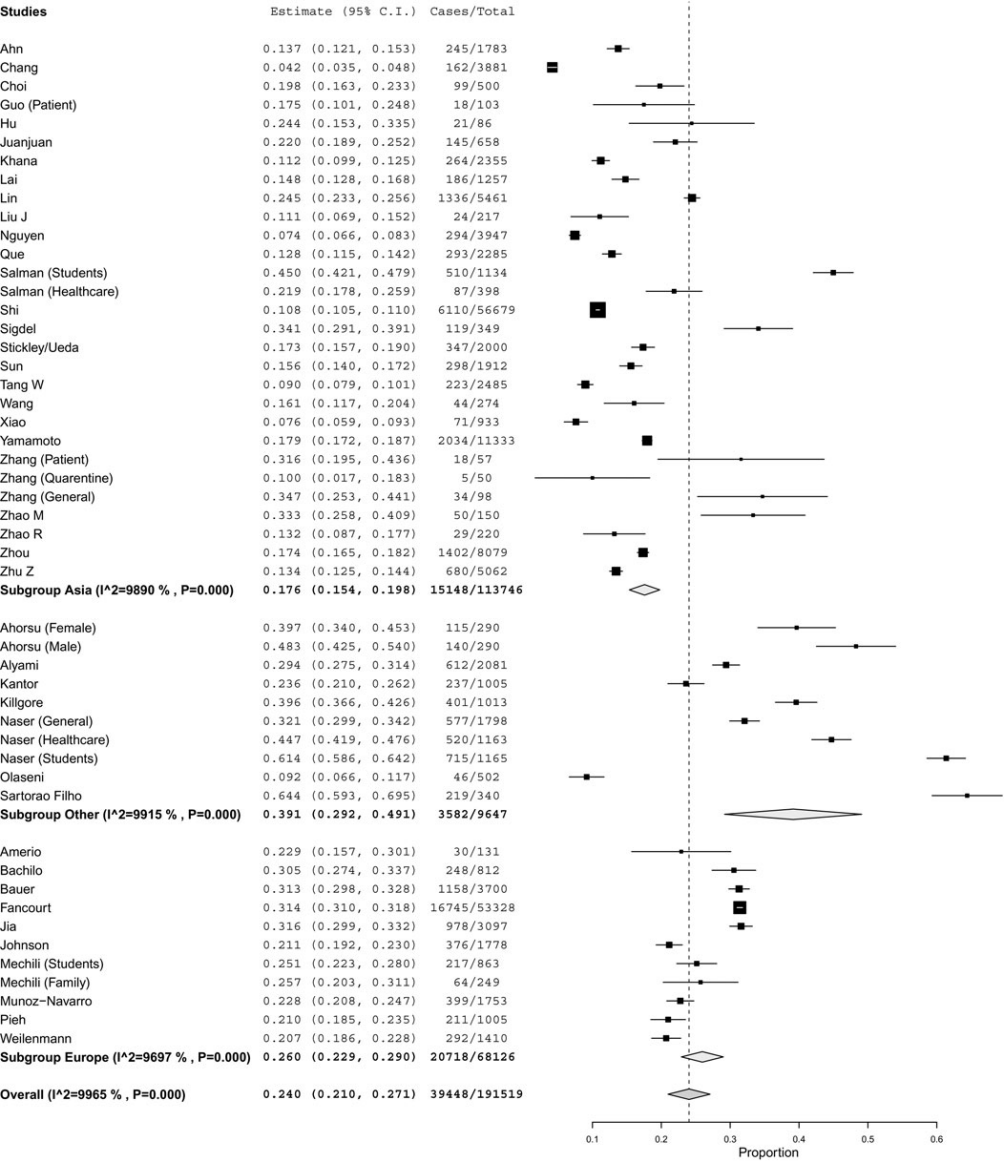
Global results of the meta-analysis for depression and a subgroup analysis by region of the world.

Figure 2 presents both the global results of the meta-analysis for depression and a subgroup analysis by region of the world (*N* = 191 519). We found a global prevalence of 24.0% [95% confidence interval (CI) 21.0–27.1%] of depression; depression was observed among 17.6% (95% CI 15.4–19.8%) in Asia, among 26.0% (95% CI 22.9–29.05%) in Europe, and among 39.1% (95% CI 29.2–49.1%) in other regions of the world. A subgroup analysis (online Supplementary Fig. S3) demonstrated that China had a lower prevalence of depression (16.2%, 95% CI 13.7–18.2%) than in other countries (29.0%, 95% CI 24.8–33.2%). Additional subgroup analyses (online Supplementary Figs S4, S5, and S6) found no significant differences by population type, country income level, or being a local study.

**Fig. 3.**
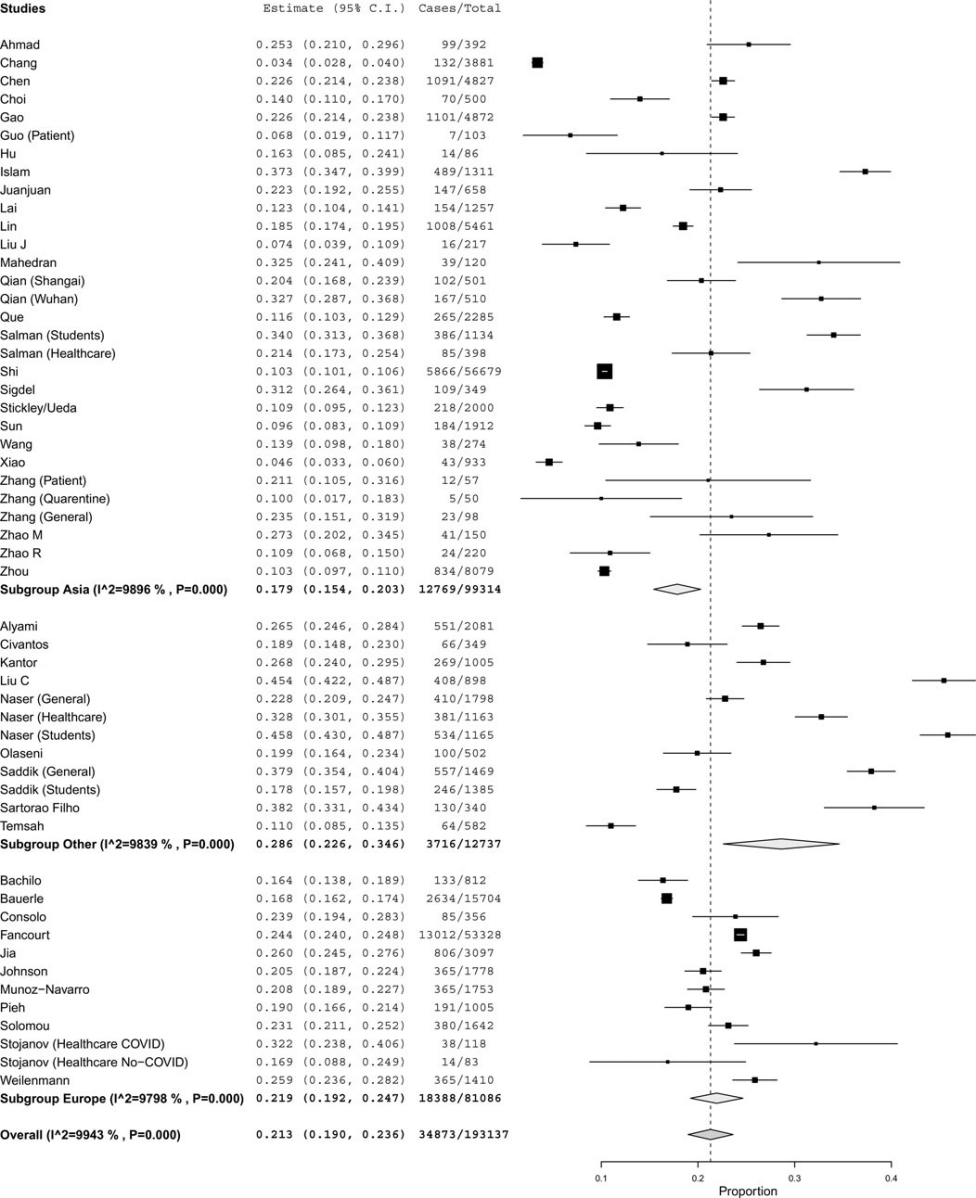
Global results of the meta-analysisfor anxiety and a subgroup analysis by region of the world.

Figure 3 presents the global results for anxiety, with a subgroup analysis by region of the world (*N* = 193 137). We found a global prevalence of anxiety of 21.3% (95% CI 19.0–23.6%). Asia had lower levels of anxiety (17.9%, 95% CI 15.4–20.3%) compared to other regions of the world (28.6%, 95% CI 22.6–34.6%). Europe did not differ from Asia and the other regions of the world. Subgroup analysis at the country-level (online Supplementary Fig. S7) showed that China had a lower prevalence of anxiety (15.5%, 95% CI 13.1–17.9%) compared to all other countries (25.6%, 95% CI 23.1–28.0%). The number of studies in each of the other countries was too restrictive to make country-specific comparisons (i.e. USA was the second country with more studies having just four studies). Further subgroup analysis (online Supplementary Figs S8, S9, and S10) found no significant differences by population type, country income level, or being a local study.

Table 1 shows the results of the meta-regression models for depression. Both in the 2- and 4-week physical distancing models, previous depression, older studies, and other region of the world than Asia/Europe were associated with depression. In addition, patient studies had a higher prevalence of depression in the 2-week physical distancing model. No significant association with physical distancing implementation measures was found in both models.

**Table 1.** Results for the meta-regression models for depression

Covariate	Coefficient	95% CI (min)	95% CI (max)	s.e.	*p*
2-week model					
School closing (2 weeks)	−0.038	−0.148	0.073	0.057	0.506
Workplace closing (2 weeks)	0.010	−0.096	0.116	0.054	0.852
Cancel public events (2 weeks)	−0.094	−0.257	0.068	0.083	0.253
Restrictions on gatherings (2 weeks)	0.016	−0.026	0.058	0.022	0.465
Close public transport (2 weeks)	0.030	−0.035	0.095	0.033	0.369
Stay-at-home requirements (2 weeks)	0.001	−0.058	0.060	0.030	0.969
Restrictions on internal movement (2 weeks)	0.039	−0.080	0.158	0.061	0.525
International travel controls (2 weeks)	−0.006	−0.032	0.019	0.013	0.626
Female	0.081	−0.072	0.233	0.078	0.302
Previous depression	**7.202**	**1.058**	**13.346**	**3.135**	**0.022**
Time	**−0.002**	**−0.004**	**−0.001**	**<0.001**	**0.003**
Population type (reference = healthcare)					
General	−0.001	−0.081	0.080	0.041	0.990
Mixed	−0.023	−0.133	0.087	0.056	0.678
Patient	**0.098**	**0.002**	**0.194**	**0.049**	**0.046**
Students	0.051	−0.028	0.131	0.041	0.207
Continent (reference = Asia)					
Europe	0.057	−0.022	0.137	0.040	0.155
Other	**0.146**	**0.061**	**0.232**	**0.044**	**<0.001**
Regional status (reference = national)					
Regional	0.063	−0.003	0.130	0.034	0.061
4-week model					
School closing (4 weeks)	−0.017	−0.152	0.118	0.069	0.804
Workplace closing (4 weeks)	−0.068	−0.185	0.049	0.060	0.257
Cancel public events (4 weeks)	−0.055	−0.202	0.091	0.075	0.458
Restrictions on gatherings (4 weeks)	0.036	−0.018	0.089	0.027	0.191
Close public transport (4 weeks)	0.028	−0.041	0.097	0.035	0.425
Stay-at-home requirements (4 weeks)	0.004	−0.075	0.083	0.040	0.915
Restrictions on internal movement (4 weeks)	0.050	−0.109	0.210	0.081	0.537
International travel controls (4 weeks)	−0.011	−0.045	0.024	0.018	0.543
Female	0.073	−0.078	0.224	0.077	0.345
Previous depression	**7.475**	**1.369**	**13.581**	**3.115**	**0.016**
Time	**−0.003**	**−0.004**	**−0.001**	**<0.001**	**0.002**
Population type (reference = healthcare)					
General	−0.020	−0.103	0.064	0.043	0.642
Mixed	−0.038	−0.148	0.072	0.056	0.493
Patient	0.088	−0.009	0.184	0.049	0.076
Students	0.046	−0.034	0.126	0.041	0.262
Continent (reference = Asia)					
Europe	0.042	−0.042	0.126	0.043	0.331
Other	**0.147**	**0.054**	**0.240**	**0.048**	**0.002**
Regional status (reference = national)					
Regional	0.059	−0.006	0.124	0.033	0.075

95% CI, 95% confidence interval; s.e., standard error.

Bold significance *p* < 0.05.

Table 2 presents the results of the meta-regression models for anxiety. Both in the 2- and 4-week physical distancing models, the closure of public transportation was associated with anxiety. Student studies had lower levels of anxiety in both models. No other significant association between physical distancing measures and depression or anxiety was found. Sensitivity analyses confirmed the results for the 2-week closure of public transportation (online Supplementary Tables S5 and S6).

**Table 2.** Results for the meta-regression models for anxiety

Covariate	Coefficient	95% CI (min)	95% CI (max)	s.e.	*p*
2-week model					
School closing (2 weeks)	0.002	−0.093	0.098	0.049	0.961
Workplace closing (2 weeks)	−0.001	−0.099	0.097	0.050	0.988
Cancel public events (2 weeks)	0.007	−0.195	0.208	0.103	0.949
Restrictions on gatherings (2 weeks)	0.022	−0.065	0.109	0.044	0.615
Close public transport (2 weeks)	**0.071**	**0.007**	**0.134**	**0.032**	**0.029**
Stay-at-home requirements (2 weeks)	−0.029	−0.096	0.038	0.034	0.399
Restrictions on internal movement (2 weeks)	−0.043	−0.173	0.086	0.066	0.512
International travel controls (2 weeks)	0.005	−0.019	0.030	0.013	0.676
Female	0.080	−0.093	0.253	0.088	0.366
Previous anxiety	1.408	−2.472	5.288	1.980	0.477
Time	−0.001	−0.002	0.001	<0.001	0.286
Population type (reference = general)					
Healthcare	−0.040	−0.107	0.026	0.034	0.237
Mixed	−0.039	−0.127	0.049	0.045	0.385
Patient	−0.017	−0.104	0.070	0.044	0.706
Students	**−0.068**	**−0.133**	**−0.003**	**0.033**	**0.041**
Continent (reference = Asia)					
Europe	0.003	−0.100	0.106	0.053	0.955
Other	0.017	−0.036	0.071	0.027	0.530
Regional status (reference = national)					
Regional	0.091	−0.017	0.199	0.055	0.100
4-week model					
School closing (4 weeks)	−0.060	−0.162	0.043	0.052	0.256
Workplace closing (4 weeks)	−0.014	−0.119	0.091	0.054	0.792
Cancel public events (4 weeks)	0.041	−0.095	0.177	0.069	0.551
Restrictions on gatherings (4 weeks)	0.032	−0.038	0.101	0.035	0.368
Close public transport (4 weeks)	**0.066**	**0.004**	**0.128**	**0.032**	**0.038**
Stay-at-home requirements (4 weeks)	−0.031	−0.104	0.043	0.038	0.414
Restrictions on internal movement (4 weeks)	−0.030	−0.166	0.106	0.069	0.669
International travel controls (4 weeks)	0.017	−0.013	0.048	0.016	0.267
Female	0.025	−0.143	0.193	0.086	0.769
Previous anxiety	1.442	−1.906	4.789	1.708	0.399
Time	−0.001	−0.003	0.000	<0.001	0.060
Population type (reference = general)					
Healthcare	−0.059	−0.122	0.004	0.032	0.067
Mixed	−0.031	−0.108	0.046	0.039	0.426
Patient	−0.005	−0.087	0.078	0.042	0.911
Students	**−0.068**	**−0.130**	**−0.006**	**0.032**	**0.033**
Continent (reference = Asia)					
Europe	−0.022	−0.115	0.072	0.048	0.649
Other	0.061	−0.028	0.151	0.046	0.179
Regional status (reference = national)					
Regional	−0.008	−0.060	0.044	0.026	0.766

95% CI, 95% confidence interval; s.e., standard error.

## Discussion

This study aimed to investigate the levels of depression and anxiety during the COVID-19 pandemic and the effect of physical distancing measures on depression and anxiety. We found high global prevalences of both depression and anxiety during the COVID-19 pandemic (24.0% and 21.3%, respectively); however, there was a wide variance in the prevalence of both anxiety and depression reported in the region- and country-level. Asia, and China especially, presented lower levels of both anxiety and depression, compared to the other countries. Closure of public transportation increased the levels of anxiety, independently of the timeframe (2 or 4 weeks post-transportation closure enactment).

Previous research has suggested that the global healthcare sector must increase the support for the prevention and early intervention of depression and anxiety secondary to COVID-19 and physical distancing measures (Galea, Merchant, & Lurie, 2020). Within the subgroup of Asian countries, the estimates of depression prevalence ranged from 15.4% to 19.8%. When comparing to the prevalence of depression in the pre-COVID-19 era, ranging from 1.3% to 3.4% (online Supplementary Table S4), these estimates are demonstrably larger after the initiation of COVID-19. The lower levels of depression found in Asian countries could be culture-dependent. Depression is more stigmatized and under-reported in this region (Yang et al., 2020). In a large cross-national study conducted by the World Mental Health Initiative a decade ago, China and Japan presented the lowest lifetime prevalences of depressive disorders (Bromet et al., 2011). Larger differences were also found for the countries in Europe (1.4–3.9% *v.* 26.0%) and other regions (2.1–4.3% *v.* 29.2%).

Similarly, the prevalence of anxiety, as reported in the subgroup of Asian countries, is greater post-COVID-19. Rates of anxiety prior to COVID-19 ranged from 2.1% to 4.1% (online Supplementary Table S4) *v.* 17.9% in the present study. Increases in anxiety can be observed in the countries classified within the countries outside Asia and Europe (2.8–7.1% *v.* 28.6%). Similar to depression, the lower anxiety levels in Asia could be culture-dependent. The social concerns of the individual could play a role in the expression of the anxiety symptoms (Hofmann, Anu Asnaani, & Hinton, 2010). Lower prevalences of anxiety are usually found in this continent as compared to data from countries in other continents (Hofmann et al., 2010; Lee et al., 2016). Among the European countries, estimates of anxiety prevalence prior to COVID were between 3.0% and 7.4% (online Supplementary Table S4) in comparison to 19.2% subsequent to the occurrence of COVID-19.

Our finding regarding the effect of public transportation closures on anxiety levels points to the importance of these systems to global populations. Our sensitivity analysis showed significant results in Europe but not in Asia. These findings could be linked to the fact that Europe has a more effective and implemented public transport network on average, making Europeans depending more on public transportation than people in Asian countries (De Gruyter, Currie, & Rose, 2017). However, what the closure of public transportation communicates in terms of the severity of the pandemic to the population may differ between Asia and Europe. We understand that anxiety could emerge as a result of two fear/worry dimensions: not being able to achieve the basic needs and/or insecurity. Depending on the setting (i.e. rural, small to large metropolitan areas), there is a significant number of individuals who do not have an alternative way of transport (i.e. car, motorcycle) and are dependent on public transportation (Pettersson & Khan, 2020). People in many different countries and cultural contexts rely on some method of public transport for getting food, clothing, education, shelter, healthcare, sanitation (Hu, Weng, Zhou, Lin, & Liu, 2019), such as transport within metropolitan areas to places of employment (Johnson, Ercolani, & Mackie, 2017). It is thus reasonable to theorize that anxious anticipatory thinking could emerge in people dependent on public transport. These are core symptoms of many anxiety disorders (Plummer, Manea, Trepel, & McMillan, 2016), which are captured by our anxiety outcome measure (GAD-7). In addition to worry regarding the reliability of public transport, anxiety could grow from an increased risk of assault and harassment resultant from fewer bystanders accessing this method of transportation (Lewis, 2018). Considering that mitigation strategies in the COVID era have involved significantly reducing the volume of passengers, the number of routes, and the means of transport available (Iacus, Natale, Santamaria, Spyratos, & Vespe, 2020), closures of these systems can work to generate excessive anxiety and worry (Kim & Gustafson-Pearce, 2016).

### Strengths and limitations

Country-level data of physical distancing measures and previous anxiety or depression are an important limitation of the present study. However, we included data from 67 different samples from 26 countries, within five global regions (Asia, Africa, America, Europe, and Middle-East), totaling almost 200 000 individuals in each meta-analysis. In addition, we used just one outcome measure per disorder (PHQ-9 and GAD-7), to avoid outcome measure bias, common in meta-analysis studies. The choice was based on these measures' popularity for assessing depressive and anxiety symptoms during the pandemic. Some studies question the suitability of GAD-7 to diagnose categorical anxiety, despite the good psychometric properties concerning the severity of symptoms (Rutter & Brown, 2017). It may not be a good screener for social anxiety disorder (Beard & Björgvinsson, 2014). Possibly, phobic anxiety disorders may be underrepresented. Unfortunately, we were not able to include age as a covariate in the meta-regression models due to a lack of descriptive data. A portion of the included samples (35.9%, *N* = 24) were not peer-reviewed. Notably, the inclusion of data from pre-print repositories could be seen as both a strength and limitation, in that the inclusion of the most recent data is of utmost importance. In addition, the association between anxiety and transport closure remained significant in the sensitivity analysis excluding the non-peer-reviewed studies. Results should be interpreted with caution. Finally, this study also did not consider comorbid anxiety-depression, although this comorbidity is prevalent.

## Conclusion

The COVID-19 pandemic, and the resulting physical distancing measures to mitigate viral spread, has impacted population mental health worldwide. Despite finding a wide variation in anxiety and depression levels across countries and regions of the world, the high prevalence of mental health disorders is a considerable concern during the COVID era. Thus, mental health outcomes should not be addressed as a delayed consequence of the COVID-19 pandemic, but rather as an ongoing and concurrent epidemic (i.e. a syndemic). We also observed an association between restrictions and closures of public transportation systems and an increase in anxiety levels. These results have important implications for policymakers. There is an urgent need for the healthcare sector to now increase the support for the prevention and early intervention of depression and anxiety.

## Supplementary material

For supplementary material accompanying this paper visit http://dx.doi.org/10.1017/S0033291721000933. click here to view supplementary material
