# Supplementary material for: Investigating the effect of national government physical distancing measures on depression and anxiety during the COVID-19 pandemic through meta-analysis and meta-regression
Source: Psychol Med. 2021 Mar 2:1–13. doi: 10.1017/S0033291721000933 (PMC7985907; doi:10.1017/S0033291721000933)

Studies	Estimate (95% C.I.)	Cases/Total
Ahn	0.137 (0.121, 0.153)	245/1783
Alyami	0.294 (0.275, 0.314)	612/2081
Bachilo	0.305 (0.274, 0.337)	248/812
Bauer	0.313 (0.298, 0.328)	1158/3700
Fancourt	0.314 (0.310, 0.318)	16745/53328
Guo (Patient)	0.175 (0.101, 0.248)	18/103
Jia	0.316 (0.299, 0.332)	978/3097
Johnson	0.211 (0.192, 0.230)	376/1778
Juanjuan	0.220 (0.189, 0.252)	145/658
Kantor	0.236 (0.210, 0.262)	237/1005
Khana	0.112 (0.099, 0.125)	264/2355
Killgore	0.396 (0.366, 0.426)	401/1013
Lai	0.148 (0.128, 0.168)	186/1257
Lin	0.245 (0.233, 0.256)	1336/5461
Liu J	0.111 (0.069, 0.152)	24/217
Munoz−Navarro	0.228 (0.208, 0.247)	399/1753
Naser (General)	0.321 (0.299, 0.342)	577/1798
Naser (Healthcare)	0.447 (0.419, 0.476)	520/1163
Naser (Students)	0.614 (0.586, 0.642)	715/1165
Nguyen	0.074 (0.066, 0.083)	294/3947
Olaseni	0.092 (0.066, 0.117)	46/502
Pieh	0.210 (0.185, 0.235)	211/1005
Que	0.128 (0.115, 0.142)	293/2285
Salman (Students)	0.450 (0.421, 0.479)	510/1134
Shi	0.108 (0.105, 0.110)	6110/56679
Sigdel	0.341 (0.291, 0.391)	119/349
Stickley/Ueda	0.173 (0.157, 0.190)	347/2000
Sun	0.156 (0.140, 0.172)	298/1912
Tang W	0.090 (0.079, 0.101)	223/2485
Wang	0.161 (0.117, 0.204)	44/274
Weilenmann	0.207 (0.186, 0.228)	292/1410
Xiao	0.076 (0.059, 0.093)	71/933
Yamamoto	0.179 (0.172, 0.187)	2034/11333
Zhao R	0.132 (0.087, 0.177)	29/220
Zhou	0.174 (0.165, 0.182)	1402/8079
**Subgroup National (I^2=9971 % , P=0.000)**	**0.225 (0.189, 0.261)**	**37507/179074**
Ahorsu (Female)	0.397 (0.340, 0.453)	115/290
Ahorsu (Male)	0.483 (0.425, 0.540)	140/290
Amerio	0.229 (0.157, 0.301)	30/131
Chang	0.042 (0.035, 0.048)	162/3881
Choi	0.198 (0.163, 0.233)	99/500
Hu	0.244 (0.153, 0.335)	21/86
Mechili (Students)	0.251 (0.223, 0.280)	217/863
Mechili (Family)	0.257 (0.203, 0.311)	64/249
Salman (Healthcare)	0.219 (0.178, 0.259)	87/398
Sartorao Filho	0.644 (0.593, 0.695)	219/340
Zhang (Patient)	0.316 (0.195, 0.436)	18/57
Zhang (Quarentine)	0.100 (0.017, 0.183)	5/50
Zhang (General)	0.347 (0.253, 0.441)	34/98
Zhao M	0.333 (0.258, 0.409)	50/150
Zhu Z	0.134 (0.125, 0.144)	680/5062
**Subgroup Regional (I^2=9899 % , P=0.000)**	**0.278 (0.213, 0.344)**	**1941/12445**
**Overall (I^2=9965 % , P=0.000)**	**0.240 (0.210, 0.271)**	**39448/191519**

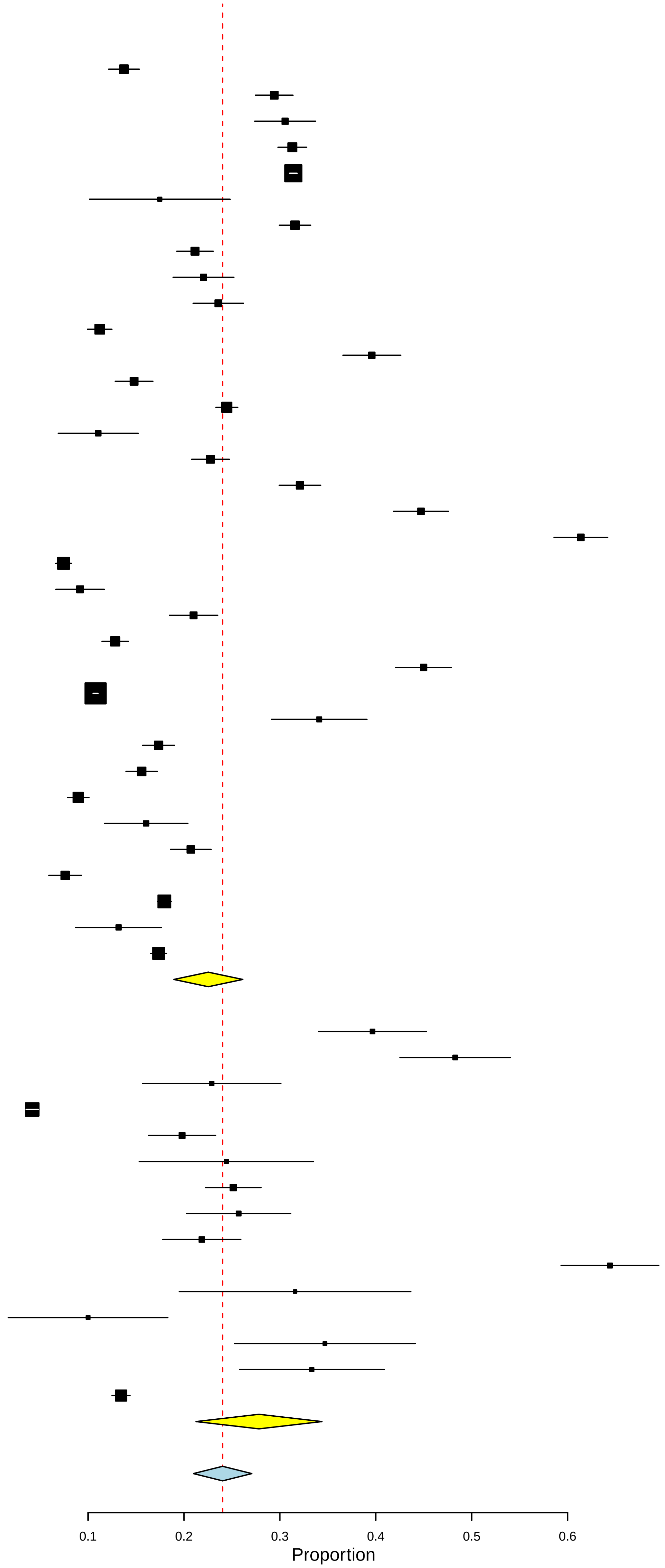

Supplement: Supplementary file 1 [file S0033291721000933sup001.zip › S0033291721000933sup001/S0033291721000933sup004.pdf]
