# Supplementary material for: Investigating the effect of national government physical distancing measures on depression and anxiety during the COVID-19 pandemic through meta-analysis and meta-regression
Source: Psychol Med. 2021 Mar 2:1–13. doi: 10.1017/S0033291721000933 (PMC7985907; doi:10.1017/S0033291721000933)

| Studies | Estimate (95% C.I.) | Cases/Total |
|---|---|---|
| Ahmad | 0.253 (0.210, 0.296) | 99/392 |
| Alyami | 0.265 (0.246, 0.284) | 551/2081 |
| Bachilo | 0.164 (0.138, 0.189) | 133/812 |
| Bauerle | 0.168 (0.162, 0.174) | 2634/15704 |
| Civantos | 0.189 (0.148, 0.230) | 66/349 |
| Consolo | 0.239 (0.194, 0.283) | 85/356 |
| Fancourt | 0.244 (0.240, 0.248) | 13012/53328 |
| Islam | 0.373 (0.347, 0.399) | 489/1311 |
| Jia | 0.260 (0.245, 0.276) | 806/3097 |
| Johnson | 0.205 (0.187, 0.224) | 365/1778 |
| Kantor | 0.268 (0.240, 0.295) | 269/1005 |
| Liu C | 0.454 (0.422, 0.487) | 408/898 |
| Munoz−Navarro | 0.208 (0.189, 0.227) | 365/1753 |
| Naser (General) | 0.228 (0.209, 0.247) | 410/1798 |
| Naser (Healthcare) | 0.328 (0.301, 0.355) | 381/1163 |
| Naser (Students) | 0.458 (0.430, 0.487) | 534/1165 |
| Olaseni | 0.199 (0.164, 0.234) | 100/502 |
| Pieh | 0.190 (0.166, 0.214) | 191/1005 |
| Saddik (General) | 0.379 (0.354, 0.404) | 557/1469 |
| Saddik (Students) | 0.178 (0.157, 0.198) | 246/1385 |
| Salman (Students) | 0.340 (0.313, 0.368) | 386/1134 |
| Salman (Healthcare) | 0.214 (0.173, 0.254) | 85/398 |
| Sartorao Filho | 0.382 (0.331, 0.434) | 130/340 |
| Sigdel | 0.312 (0.264, 0.361) | 109/349 |
| Solomou | 0.231 (0.211, 0.252) | 380/1642 |
| Stickley/Ueda | 0.109 (0.095, 0.123) | 218/2000 |
| Stojanov (Healthcare COVID) | 0.322 (0.238, 0.406) | 38/118 |
| Stojanov (Healthcare No−COVID) | 0.169 (0.088, 0.249) | 14/83 |
| Temsah | 0.110 (0.085, 0.135) | 64/582 |
| Weilenmann | 0.259 (0.236, 0.282) | 365/1410 |
| **Subgroup Other (I^2=9843 % , P=0.000)** | **0.256 (0.231, 0.280)** | **23490/99407** |
| Chang | 0.034 (0.028, 0.040) | 132/3881 |
| Chen | 0.226 (0.214, 0.238) | 1091/4827 |
| Choi | 0.140 (0.110, 0.170) | 70/500 |
| Gao | 0.226 (0.214, 0.238) | 1101/4872 |
| Guo (Patient) | 0.068 (0.019, 0.117) | 7/103 |
| Hu | 0.163 (0.085, 0.241) | 14/86 |
| Juanjuan | 0.223 (0.192, 0.255) | 147/658 |
| Lai | 0.123 (0.104, 0.141) | 154/1257 |
| Lin | 0.185 (0.174, 0.195) | 1008/5461 |
| Liu J | 0.074 (0.039, 0.109) | 16/217 |
| Mahedran | 0.325 (0.241, 0.409) | 39/120 |
| Qian (Shangai) | 0.204 (0.168, 0.239) | 102/501 |
| Qian (Wuhan) | 0.327 (0.287, 0.368) | 167/510 |
| Que | 0.116 (0.103, 0.129) | 265/2285 |
| Shi | 0.103 (0.101, 0.106) | 5866/56679 |
| Sun | 0.096 (0.083, 0.109) | 184/1912 |
| Wang | 0.139 (0.098, 0.180) | 38/274 |
| Xiao | 0.046 (0.033, 0.060) | 43/933 |
| Zhang (Patient) | 0.211 (0.105, 0.316) | 12/57 |
| Zhang (Quarentine) | 0.100 (0.017, 0.183) | 5/50 |
| Zhang (General) | 0.235 (0.151, 0.319) | 23/98 |
| Zhao M | 0.273 (0.202, 0.345) | 41/150 |
| Zhao R | 0.109 (0.068, 0.150) | 24/220 |
| Zhou | 0.103 (0.097, 0.110) | 834/8079 |
| **Subgroup China (I^2=9885 % , P=0.000)** | **0.155 (0.131, 0.179)** | **11383/93730** |
| **Overall (I^2=9943 % , P=0.000)** | **0.213 (0.190, 0.236)** | **34873/193137** |

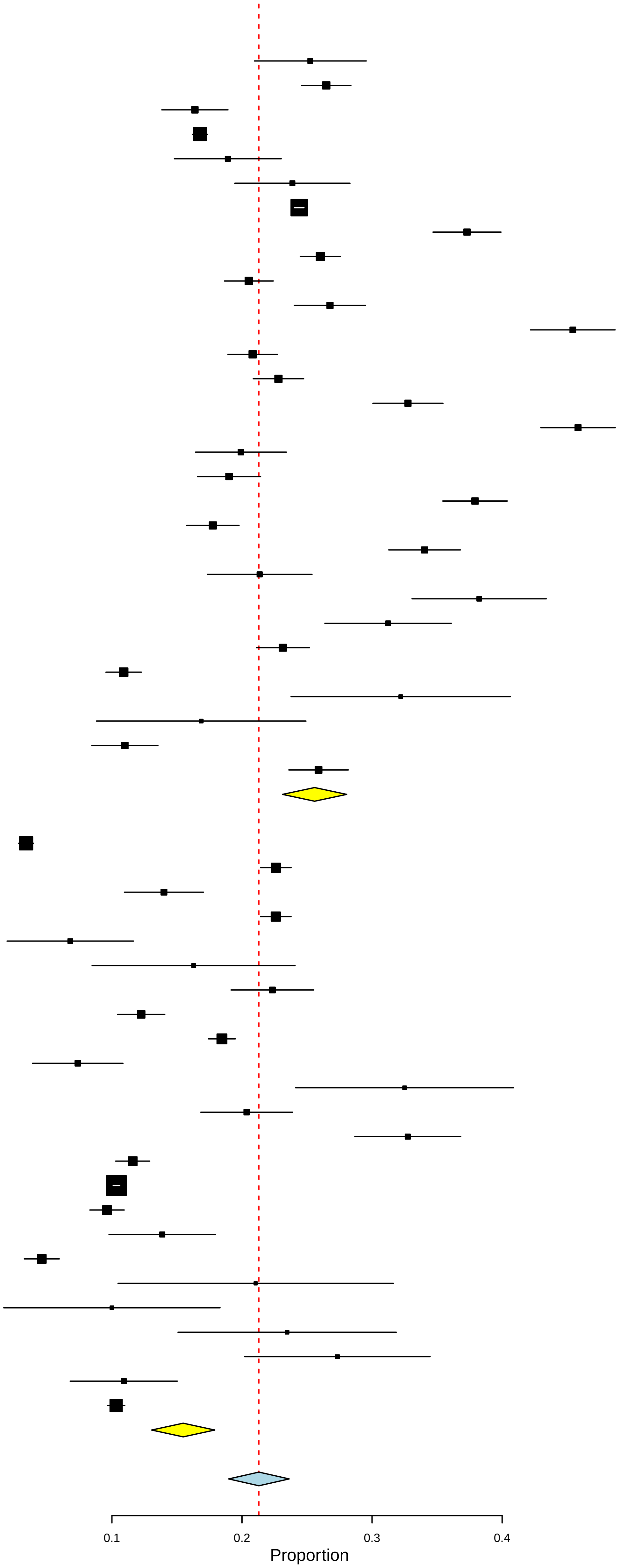

Supplement: Supplementary file 1 [file S0033291721000933sup001.zip › S0033291721000933sup001/S0033291721000933sup005.pdf]
