# Supplementary material for: Investigating the effect of national government physical distancing measures on depression and anxiety during the COVID-19 pandemic through meta-analysis and meta-regression
Source: Psychol Med. 2021 Mar 2:1–13. doi: 10.1017/S0033291721000933 (PMC7985907; doi:10.1017/S0033291721000933)
Supplement: Supplementary file 1 [file S0033291721000933sup001.zip › S0033291721000933sup001/S0033291721000933sup007.pdf]

Table S4. Previous prevalence of depression and anxiety based on the most recent published database of the Global Burden of Disease Study (GBD 2017 Disease and Injury Incidence and Prevalence Collaborators, 2018)

Author	Country	Region	PHQ-9 ≧ 10 (%)	GAD-7 ≧ 10 (%)	Depression Cases* (2017)	Anxiety Cases* (2017)	Country Population* (2017)	Depression Prevalence (2017)	Anxiety Prevalence (2017)
Ahmad	India	Asia	N.A.	25.25	30017	44873	1,338,659	2.24%	3.35%
Ahn	Korea	Asia	13.74	N.A.	1222	2002	51,362	2.38%	3.90%
Ahorsu (Female)	Iran	Middle-East	39.7	N.A.	3461	5754	80,674	4.29%	7.13%
Ahorsu (Male)	Iran	Middle-East	48.3	N.A.	3461	5754	80,674	4.29%	7.13%
Alyami	Saudi Arabia	Middle-East	29.4	26.5	1257	1735	33,099	3.80%	5.24%
Amerio	Italy	Europe	22.9	N.A.	1453	3379	60,537	2.40%	5.58%
Bachilo	Russia	Europe	30.54	16.38	3838	4398	144,497	2.66%	3.04%
Bauer	Germany	Europe	31.3	N.A.	2043	5265	82,657	2.47%	6.37%
Bauerle	Germany	Europe	N.A.	16.77	2043	5265	82,657	2.47%	6.37%
Chang	China	Asia	4.18	3.41	28672	44745	1,386,395	2.07%	3.23%
Chen	China	Asia	N.A.	22.6	28672	44745	1,386,395	2.07%	3.23%
Choi	China	Asia	19.8	14	28672	44745	1,386,395	2.07%	3.23%
Civantos	USA	America	N.A.	18.9	9611	20965	324,986	2.96%	6.45%
Consolo	Italy	Europe	N.A.	23.9	1453	3379	60,537	2.40%	5.58%
Fancourt	UK	Europe	31.4	24.4	1809	2956	66,059	2.74%	4.47%
Gao	China	Asia	N.A.	22.6	28672	44745	1,386,395	2.07%	3.23%
Guo (Patient)	China	Asia	17.5	6.8	28672	44745	1,386,395	2.07%	3.23%
Hu	China	Asia	24.7	16.47	28672	44745	1,386,395	2.07%	3.23%
Islam	Bangladesh	Asia	N.A.	37.3	4294	6575	159,671	2.69%	4.12%
Jia	UK	Europe	31.57	26.02	1809	2956	66,059	2.74%	4.47%
Johnson	Norway	Europe	21.14	20.52	127	388	5,277	2.40%	7.36%
Juanjuan	China	Asia	22.03	22.34	28672	44745	1,386,395	2.07%	3.23%
Kantor	USA	America	23.6	26.8	9611	20965	324,986	2.96%	6.45%
Kha.	India	Asia	11.23	N.A.	30017	44873	1,338,659	2.24%	3.35%
Killgore	USA	America	39.59	N.A.	9611	20965	324,986	2.96%	6.45%
Lai	China	Asia	14.79	12.25	28672	44745	1,386,395	2.07%	3.23%
Lin	China	Asia	24.46	18.46	28672	44745	1,386,395	2.07%	3.23%
Liu C	USA	America	N.A.	45.4	9611	20965	324,986	2.96%	6.45%
Liu J	China	Asia	11.05	7.37	28672	44745	1,386,395	2.07%	3.23%
Mahedran	China	Asia	N.A.	32.5	28672	44745	1,386,395	2.07%	3.23%
Mechili (Students)	Albania	Europe	25.14	N.A.	41	98	2,873	1.42%	3.40%
Mechili (Family)	Albania	Europe	25.6	N.A.	41	98	2,873	1.42%	3.40%
Munoz-.varro	Spain	Europe	22.76	20.8	1824	2397	46,593	3.91%	5.14%

Naser (General)	Jordan	Middle-East	32.09	22.8	244	495	9,779	2.49%	5.06%
Naser (Healthcare)	Jordan	Middle-East	44.71	32.76	244	495	9,779	2.49%	5.06%
Naser (Students)	Jordan	Middle-East	61.37	45.83	244	495	9,779	2.49%	5.06%
Nguyen	Vietnam	Asia	7.44	N.A.	1269	2006	94,597	1.34%	2.12%
Olaseni	Nigeria	Africa	9.16	19.92	4080	5341	190,873	2.14%	2.80%
Pieh	Austria	Europe	20.99	19	168	460	8,798	1.91%	5.23%
Qian (Shangai)	China	Asia	N.A.	20.35	28672	44745	1,386,395	2.07%	3.23%
Qian (Wuhan)	China	Asia	N.A.	32.74	28672	44745	1,386,395	2.07%	3.23%
Que	China	Asia	12.82	11.6	28672	44745	1,386,395	2.07%	3.23%
Saddik (General)	UAE	Middle-East	N.A.	37.91	226	458	9,487	2.38%	4.82%
Saddik (Students)	UAE	Middle-East	N.A.	17.76	226	458	9,487	2.38%	4.82%
Salman (Students)	Pakistan	Asia	45	34	3559	7345	207,897	1.71%	3.53%
Salman (Healthcare)	Pakistan	Asia	21.8	21.3	3559	7345	207,897	1.71%	3.53%
Sartorao Filho	Brazil	America	64.41	38.23	5031	13197	207,834	2.42%	6.35%
Shi	China	Asia	10.78	10.35	28672	44745	1,386,395	2.07%	3.23%
Sigdel	Nepal	Asia	34.1	31.2	948	1100	27,627	3.43%	3.98%
Solomou	Cyprus	Europe	N.A.	23.14	24	66	1,180	2.05%	5.61%
Stickley/Ueda	Japan	Asia	17.35	10.9	2992	4305	126,786	2.36%	3.40%
Stojanov (Healthcare/COVID)	Serbia	Europe	N.A.	31.8	182	318	7,021	2.59%	4.53%
Stojanov (Healthcare/No-COVID)	Serbia	Europe	N.A.	16.4	182	318	7,021	2.59%	4.53%
Sun	China	Asia	15.58	9.62	28672	44745	1,386,395	2.07%	3.23%
Tang W	China	Asia	8.97	N.A.	28672	44745	1,386,395	2.07%	3.23%
Temsah	Saudi Arabia	Middle-East	N.A.	10.99	1257	1735	33,099	3.80%	5.24%
Wang	China	Asia	16.1	13.9	28672	44745	1,386,395	2.07%	3.23%
Weilenmann	Switzerland	Europe	20.7	25.88	204	446	8,452	2.41%	5.28%
Xiao	China	Asia	7.6	4.6	28672	44745	1,386,395	2.07%	3.23%
Yamamoto	Japan	Asia	17.95	N.A.	2992	4305	126,786	2.36%	3.40%
Zhang (Patient)	China	Asia	31.57	21.05	28672	44745	1,386,395	2.07%	3.23%
Zhang (Quarentine)	China	Asia	10	10	28672	44745	1,386,395	2.07%	3.23%
Zhang (General)	China	Asia	34.69	23.46	28672	44745	1,386,395	2.07%	3.23%
Zhao M	China	Asia	33.33	27.33	28672	44745	1,386,395	2.07%	3.23%
Zhao R	China	Asia	13.18	10.9	28672	44745	1,386,395	2.07%	3.23%
Zhou	China	Asia	17.35	10.32	28672	44745	1,386,395	2.07%	3.23%
Zhu Z	China	Asia	13.44	N.A.	28672	44745	1,386,395	2.07%	3.23%

in thousands
