# Supplementary material for: Investigating the effect of national government physical distancing measures on depression and anxiety during the COVID-19 pandemic through meta-analysis and meta-regression
Source: Psychol Med. 2021 Mar 2:1–13. doi: 10.1017/S0033291721000933 (PMC7985907; doi:10.1017/S0033291721000933)
Supplement: Supplementary file 1 [file S0033291721000933sup001.zip › S0033291721000933sup001/S0033291721000933sup008.pdf]

Table S5. Results of the sensitivity analysis (only Asian, European and Peer-reviewed studies) investigating the association between transport closure and anxiety.

**Covariate**	**Coefficient**	**95%CI (min)**	**95%CI (max)**	**SE**	**p**
**2-week model**					
*Only Asian studies**	0.039	-0.064	0.143	0.053	0.453
*Only European Studies***	0.092	0.036	0.148	0.029	0.001
*Only peer-reviewed studies****	0.089	0.006	0.173	0.043	0.037
**4-week model**					
*Only Asian studies**	0.035	-0.077	0.146	0.057	0.540
*Only European Studies***	0.078	0.017	0.139	0.031	0.013
*Only peer-reviewed studies ****	0.078	0.004	0.153	0.038	0.040

95%CI = 95% Confidence Interval; SE = Standard error

** Adjusted for school closing, workplace closing, cancel public events, restrictions on gatherings, close public transport, stay-at-home requirements, restrictions on internal movement, international travel controls, female, previous anxiety, time, population type, and regional status*

*** Adjusted for school closing, workplace closing, cancel public events, restrictions on gatherings, close public transport, stay-at-home requirements, restrictions on internal movement, international travel controls, previous anxiety and time. The model did not reach convergence fo female, population type, and regional status*

** Adjusted for school closing, workplace closing, cancel public events, restrictions on gatherings, close public transport, stay-at-home requirements, restrictions on internal movement, international travel controls, female, previous anxiety, time, region, population type, and regional status*
