# Supplementary material for: Investigating the effect of national government physical distancing measures on depression and anxiety during the COVID-19 pandemic through meta-analysis and meta-regression
Source: Psychol Med. 2021 Mar 2:1–13. doi: 10.1017/S0033291721000933 (PMC7985907; doi:10.1017/S0033291721000933)

Studies	Estimate (95% C.I.)	Cases/Total
Ahmad	0.253 (0.210, 0.296)	99/392
Alyami	0.265 (0.246, 0.284)	551/2081
Bachilo	0.164 (0.138, 0.189)	133/812
Bauerle	0.168 (0.162, 0.174)	2634/15704
Chen	0.226 (0.214, 0.238)	1091/4827
Civantos	0.189 (0.148, 0.230)	66/349
Fancourt	0.244 (0.240, 0.248)	13012/53328
Gao	0.226 (0.214, 0.238)	1101/4872
Guo (Patient)	0.068 (0.019, 0.117)	7/103
Islam	0.373 (0.347, 0.399)	489/1311
Jia	0.260 (0.245, 0.276)	806/3097
Johnson	0.205 (0.187, 0.224)	365/1778
Juanjuan	0.223 (0.192, 0.255)	147/658
Kantor	0.268 (0.240, 0.295)	269/1005
Lai	0.123 (0.104, 0.141)	154/1257
Lin	0.185 (0.174, 0.195)	1008/5461
Liu C	0.454 (0.422, 0.487)	408/898
Liu J	0.074 (0.039, 0.109)	16/217
Munoz−Navarro	0.208 (0.189, 0.227)	365/1753
Naser (General)	0.228 (0.209, 0.247)	410/1798
Naser (Healthcare)	0.328 (0.301, 0.355)	381/1163
Naser (Students)	0.458 (0.430, 0.487)	534/1165
Olaseni	0.199 (0.164, 0.234)	100/502
Pieh	0.190 (0.166, 0.214)	191/1005
Que	0.116 (0.103, 0.129)	265/2285
Saddik (General)	0.379 (0.354, 0.404)	557/1469
Saddik (Students)	0.178 (0.157, 0.198)	246/1385
Salman (Students)	0.340 (0.313, 0.368)	386/1134
Shi	0.103 (0.101, 0.106)	5866/56679
Sigdel	0.312 (0.264, 0.361)	109/349
Solomou	0.231 (0.211, 0.252)	380/1642
Stickley/Ueda	0.109 (0.095, 0.123)	218/2000
Sun	0.096 (0.083, 0.109)	184/1912
Wang	0.139 (0.098, 0.180)	38/274
Weilenmann	0.259 (0.236, 0.282)	365/1410
Xiao	0.046 (0.033, 0.060)	43/933
Zhao R	0.109 (0.068, 0.150)	24/220
Zhou	0.103 (0.097, 0.110)	834/8079
**Subgroup National (I^2=9949 % , P=0.000)**	**0.213 (0.187, 0.239)**	**33852/185307**
Chang	0.034 (0.028, 0.040)	132/3881
Choi	0.140 (0.110, 0.170)	70/500
Consolo	0.239 (0.194, 0.283)	85/356
Hu	0.163 (0.085, 0.241)	14/86
Mahedran	0.325 (0.241, 0.409)	39/120
Qian (Shangai)	0.204 (0.168, 0.239)	102/501
Qian (Wuhan)	0.327 (0.287, 0.368)	167/510
Salman (Healthcare)	0.214 (0.173, 0.254)	85/398
Sartorao Filho	0.382 (0.331, 0.434)	130/340
Stojanov (Healthcare COVID)	0.322 (0.238, 0.406)	38/118
Stojanov (Healthcare No−COVID)	0.169 (0.088, 0.249)	14/83
Temsah	0.110 (0.085, 0.135)	64/582
Zhang (Patient)	0.211 (0.105, 0.316)	12/57
Zhang (Quarentine)	0.100 (0.017, 0.183)	5/50
Zhang (General)	0.235 (0.151, 0.319)	23/98
Zhao M	0.273 (0.202, 0.345)	41/150
**Subgroup Regional (I^2=9804 % , P=0.000)**	**0.214 (0.150, 0.279)**	**1021/7830**
**Overall (I^2=9943 % , P=0.000)**	**0.213 (0.190, 0.236)**	**34873/193137**

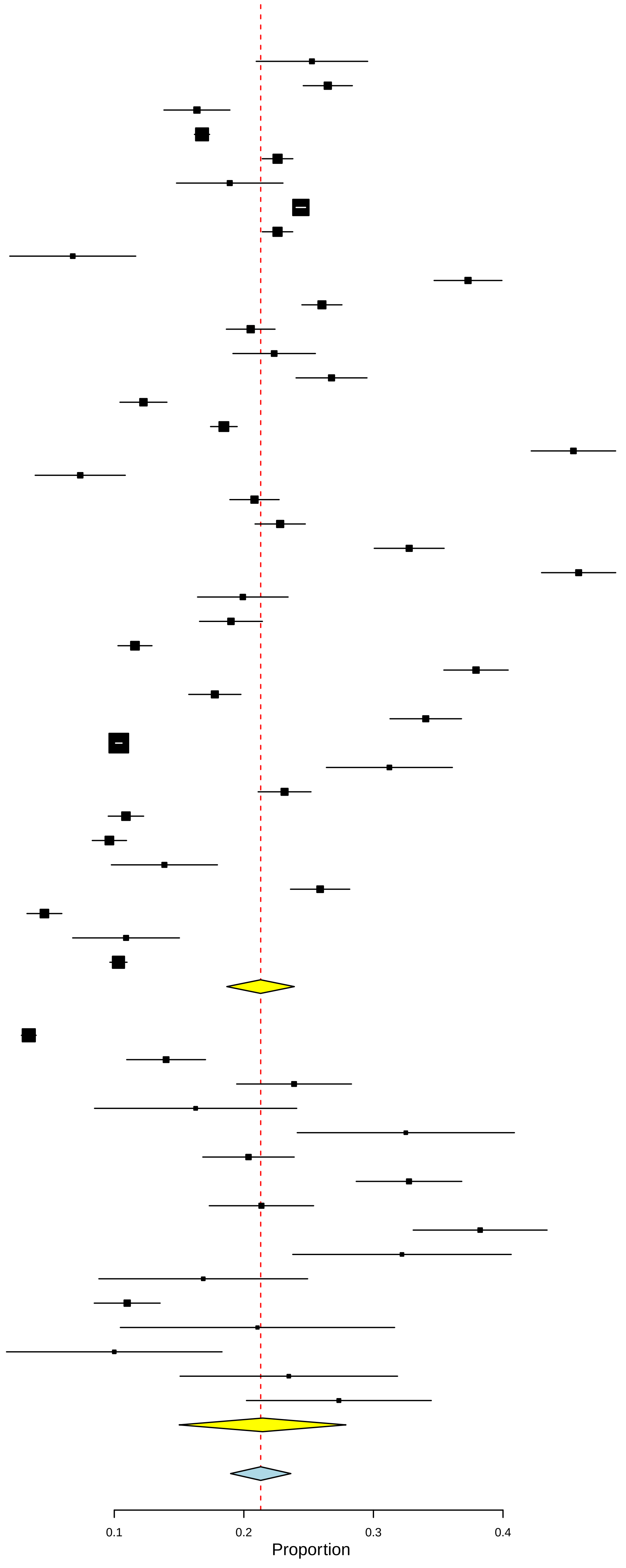

Supplement: Supplementary file 1 [file S0033291721000933sup001.zip › S0033291721000933sup001/S0033291721000933sup009.pdf]
