# Supplementary material for: Investigating the effect of national government physical distancing measures on depression and anxiety during the COVID-19 pandemic through meta-analysis and meta-regression
Source: Psychol Med. 2021 Mar 2:1–13. doi: 10.1017/S0033291721000933 (PMC7985907; doi:10.1017/S0033291721000933)
Supplement: Supplementary file 1 [file S0033291721000933sup001.zip › S0033291721000933sup001/S0033291721000933sup011.pdf]

Table S1. Main findings from the included studies.

Author	Country	n	Population Type	Initial Date	End Date	Length	Time	Mean Age/ Median	SD/ IQR	Min Age	Max Age	Female	Represent	Regional	PHQ-9 ≧ 10 (n)	PHQ-9 ≧ 10 (%)	GAD-7 ≧ 10 (n)	GAD-7 ≧ 10 (%)
Ahmad	India	392	General	3/29/20	4/12/20	14	125	30.30	9.28	18	71	47%	No	No	N.A.	N.A.	99	25.25
Ahn	Korea	1783	Healthcare	4/20/20	4/30/20	10	103	N.A.	N.A.	20	65	76%	No	No	245	13.74	N.A.	N.A.
Ahorsu (Female)	Iran	290	General	3/7/20	4/21/20	45	147	29.24	5.84	N.A.	N.A.	100%	No	Yes	115	39.7	N.A.	N.A.
Ahorsu (Male)	Iran	290	General	3/7/20	4/21/20	45	147	33.61	6.36	N.A.	N.A.	0%	No	Yes	140	48.3	N.A.	N.A.
Alyami	Saudi Arabia	2081	General	3/27/20	4/27/20	31	127	N.A.	N.A.	18	N.A.	33%	No	No	612	29.4	551	26.5
Amerio	Italy	131	Healthcare	3/15/20	4/15/20	31	139	52.31	12.24	N.A.	N.A.	48%	No	Yes	30	22.9	N.A.	N.A.
Bachilo	Russia	812	Mixed	4/21/20	5/18/20	27	102	N.A.	N.A.	20	N.A.	81%	No	No	248	30.54	133	16.38
Bauer	Germany	3700	General	4/8/20	4/26/20	18	115	33.13	11.73	18	85	79%	No	No	1158	31.3	N.A.	N.A.
Bauerle	Germany	15704	General	3/10/20	5/5/20	56	144	N.A.	N.A.	18	N.A.	71%	No	No	N.A.	N.A.	2634	16.77
Chang	China	3881	Students	1/31/20	2/3/20	3	183	20.00	3.00	N.A.	N.A.	63%	No	Yes	162	4.18	132	3.41
Chen	China	4827	General	1/31/20	2/2/20	2	183	32.30	10.00	18	85	68%	No	No	N.A.	N.A.	1091	22.6
Choi	China	500	General	4/24/20	5/3/20	9	99	47.26	15.82	N.A.	N.A.	55%	No	Yes	99	19.8	70	14
Civantos	USA	349	Healthcare	4/14/20	4/25/20	11	109	N.A.	N.A.	26	N.A.	39%	No	No	N.A.	N.A.	66	18.9
Consolo	Italy	356	Healthcare	4/2/20	4/21/20	19	121	N.A.	N.A.	N.A.	N.A.	40%	No	Yes	N.A.	N.A.	85	23.9
Fancourt	UK	53328	General	3/21/20	5/10/20	50	133	N.A.	N.A.	N.A.	N.A.	48%	No	No	16745	31.4	13012	24.4
Gao	China	4872	General	1/31/20	2/2/20	2	183	N.A.	N.A.	18	N.A.	68%	No	No	N.A.	N.A.	1101	22.6
Guo (Patient)	China	103	Patient	2/10/20	2/28/20	18	173	42.50	12.53	18	75	43%	No	No	18	17.5	7	6.8
Hu	China	86	Patient	3/7/20	3/24/20	17	147	N.A.	N.A.	N.A.	N.A.	50%	No	Yes	21	24.7	14	16.47
Islam	Bangladesh	1311	General	3/29/20	4/6/20	8	125	23.54	4.97	13	63	40%	No	No	N.A.	N.A.	489	37.3
Jia	UK	3097	Mixed	4/3/20	4/30/20	27	120	44.00	15.00	18	N.A.	85%	No	No	978	31.57	806	26.02
Johnson	Norway	1778	Mixed	3/31/20	4/7/20	7	123	N.A.	N.A.	19	N.A.	85%	No	No	376	21.14	365	20.52
Juanjuan	China	658	Patient	2/16/20	2/19/20	3	167	N.A.	N.A.	N.A.	N.A.	100%	No	No	145	22.03	147	22.34
Kantor	USA	1005	General	3/29/20	3/31/20	2	125	45.00	16.00	18	N.A.	51%	Yes	No	237	23.6	269	26.8
Khana	India	2355	Healthcare	4/15/20	4/19/20	4	108	42.50	12.05	25	82	43%	No	No	264	11.23	N.A.	N.A.
Killgore	USA	1013	General	4/9/20	4/10/20	1	114	N.A.	N.A.	18	35	44%	Yes	No	401	39.59	N.A.	N.A.
Lai	China	1257	Healthcare	1/29/20	2/3/20	5	185	N.A.	N.A.	18	N.A.	77%	No	No	186	14.79	154	12.25
Lin	China	5461	General	2/5/20	2/23/20	18	178	N.A.	N.A.	N.A.	N.A.	70%	No	No	1336	24.46	1008	18.46
Liu C	USA	898	Mixed	4/13/20	5/19/20	36	110	24.50	N.A.	18	30	81%	No	No	N.A.	N.A.	408	45.4
Liu J	China	217	Students	2/23/20	4/2/20	39	160	21.70	1.70	18	27	59%	No	No	24	11.05	16	7.37
Mahedran	China	120	Healthcare	1/24/20	2/13/20	20	190	35.00	N.A.	19	63	73%	No	Yes	N.A.	N.A.	39	32.5
Mechili (Students)	Albania	863	Students	3/30/20	4/9/20	10	124	N.A.	N.A.	18	N.A.	89%	No	Yes	217	25.14	N.A.	N.A.
Mechili (Family)	Albania	249	General	3/30/20	4/9/20	10	124	36.67	4.65	18	85	71%	No	Yes	64	25.6	N.A.	N.A.
Munoz-Navarro	Spain	1753	General	3/25/20	4/25/20	31	129	40.40	12.90	N.A.	N.A.	77%	No	No	399	22.76	365	20.8
Naser (General)	Jordan	1798	General	3/22/20	3/28/20	6	132	N.A.	N.A.	18	N.A.	64%	No	No	577	32.09	410	22.8
Naser (Healthcare)	Jordan	1163	Healthcare	3/22/20	3/28/20	6	132	N.A.	N.A.	18	N.A.	56%	No	No	520	44.71	381	32.76
Naser (Students)	Jordan	1165	Students	3/22/20	3/28/20	6	132	N.A.	N.A.	18	N.A.	54%	No	No	715	61.37	534	45.83
Nguyen	Vietnam	3947	Patient	2/14/20	3/2/20	17	169	44.40	17.00	18	85	56%	No	No	294	7.44	N.A.	N.A.
Olaseni	Nigeria	502	General	3/20/20	4/12/20	23	134	28.75	8.17	18	78	45%	No	No	46	9.16	100	19.92
Pieh	Austria	1005	General	4/17/20	4/30/20	13	106	N.A.	N.A.	18	N.A.	53%	Yes	No	211	20.99	191	19
Qian (Shangai)	China	501	General	2/1/20	2/10/20	9	182	N.A.	N.A.	18	N.A.	49%	No	Yes	N.A.	N.A.	102	20.35
Qian (Wuhan)	China	510	General	2/1/20	2/10/20	9	182	N.A.	N.A.	18	N.A.	50%	No	Yes	N.A.	N.A.	167	32.74
Que	China	2285	Healthcare	2/16/20	2/23/20	7	167	31.06	6.99	17	64	69%	No	No	293	12.82	265	11.6
Saddik (General)	UAE	1469	General	3/24/20	5/15/20	52	130	N.A.	N.A.	18	N.A.	83%	No	No	N.A.	N.A.	557	37.91
Saddik (Students)	UAE	1385	Students	3/11/20	3/21/20	10	143	20.50	2.30	N.A.	N.A.	72%	No	No	N.A.	N.A.	246	17.76
Salman (Students)	Pakistan	1134	Students	4/1/20	5/31/20	60	122	21.70	3.50	18	N.A.	71%	No	No	510	45	386	34
Salman (Healthcare)	Pakistan	398	Healthcare	4/15/20	5/20/20	35	108	28.67	4.15	N.A.	N.A.	54%	No	Yes	87	21.8	85	21.3

Sartorao Filho	Brazil	340	Students	5/18/20	5/19/20	1	75	N.A.	N.A.	18	N.A.	74%	No	Yes	219	64.41	130	38.23
Shi	China	56679	General	2/28/20	3/11/20	12	155	35.97	8.22	18	N.A.	52%	No	No	6110	10.78	5866	10.35
Sigdel	Nepal	349	General	4/6/20	4/16/20	10	117	27.80	6.60	18	N.A.	46%	No	No	119	34.1	109	31.2
Solomou	Cyprus	1642	Mixed	4/3/20	4/9/20	6	120	N.A.	N.A.	18	N.A.	72%	No	No	N.A.	N.A.	380	23.14
Stickley/Ueda	Japan	2000	General	4/16/20	4/18/20	2	107	N.A.	N.A.	N.A.	N.A.	50%	Yes	No	347	17.35	218	10.9
Stojanov (Healthcare/COVID)	Serbia	118	Healthcare	4/20/20	4/20/20	0	103	39.10	7.30	N.A.	N.A.	66%	No	Yes	N.A.	N.A.	38	31.8
Stojanov (Healthcare/No-COVID)	Serbia	83	Healthcare	4/20/20	4/20/20	0	103	42.50	9.70	N.A.	N.A.	66%	No	Yes	N.A.	N.A.	14	16.4
Sun	China	1912	Students	3/20/20	4/10/20	21	134	20.28	2.10	18	49	70%	No	No	298	15.58	184	9.62
Tang	China	2485	Students	2/20/20	2/27/20	7	163	19.81	1.55	16	27	61%	No	No	223	8.97	N.A.	N.A.
Temsah	Saudi Arabia	582	Healthcare	2/5/20	2/16/20	11	178	36.02	8.50	N.A.	N.A.	75%	No	Yes	N.A.	N.A.	64	10.99
Wang	China	274	Healthcare	2/26/20	3/3/20	6	157	37.00	N.A.	22	64	77%	No	No	44	16.1	38	13.9
Weilenmann	Switzerland	1410	Healthcare	3/28/20	4/4/20	7	126	36.45	12.61	N.A.	N.A.	66%	No	No	292	20.7	365	25.88
Xiao	China	933	Students	2/4/20	2/12/20	8	179	N.A.	N.A.	17	N.A.	70%	No	No	71	7.6	43	4.6
Yamamoto	Japan	11333	General	5/11/20	5/12/20	1	82	46.30	14.60	18	89	52%	No	No	2034	17.95	N.A.	N.A.
Zhang (Patient)	China	57	Patient	2/15/20	2/29/20	14	168	46.90	15.37	N.A.	N.A.	49%	No	Yes	18	31.57	12	21.05
Zhang (Quarentine)	China	50	Mixed	2/15/20	2/29/20	14	168	36.20	10.91	N.A.	N.A.	46%	No	Yes	5	10	5	10
Zhang (General)	China	98	General	2/15/20	2/29/20	14	168	29.60	12.69	N.A.	N.A.	65%	No	Yes	34	34.69	23	23.46
Zhao M	China	150	Patient	2/3/20	2/10/20	7	180	N.A.	N.A.	15	N.A.	41%	No	Yes	50	33.33	41	27.33
Zhao R	China	220	Mixed	2/10/20	2/15/20	5	173	40.00	10.00	N.A.	N.A.	83%	No	No	29	13.18	24	10.9
Zhou	China	8079	Students	3/8/20	3/15/20	7	146	16.00	N.A.	12	18	54%	No	No	1402	17.35	834	10.32
Zhu	China	5062	Healthcare	2/8/20	2/10/20	2	175	N.A.	N.A.	19	N.A.	85%	No	Yes	680	13.44	N.A.	N.A.
