# Supplementary material for: Investigating the effect of national government physical distancing measures on depression and anxiety during the COVID-19 pandemic through meta-analysis and meta-regression
Source: Psychol Med. 2021 Mar 2:1–13. doi: 10.1017/S0033291721000933 (PMC7985907; doi:10.1017/S0033291721000933)
Supplement: Supplementary file 1 [file S0033291721000933sup001.zip › S0033291721000933sup001/S0033291721000933sup012.pdf]

Table S2. Quality Assessment Results.

Author	Country	Sample Framework	Sample Process	Sample Size	Description	Coverage	Validity	Reliability	Statistical Analysis	Response Rate	Quality Score
Ahmad	India	Yes	No	No	Yes	Yes	Yes	Yes	Yes	No	6
Ahn	Korea	Yes	Yes	Yes	No	Yes	Yes	Yes	Yes	No	7
Ahorsu (Female)	Iran	Yes	Yes	No	Yes	Yes	Yes	Yes	No	Yes	7
Ahorsu (Male)	Iran	Yes	Yes	No	Yes	Yes	Yes	Yes	No	Yes	7
Alyami	Saudi Arabia	Yes	No	Yes	Yes	Yes	Yes	Yes	Yes	No	7
Amerio	Italy	Yes	No	No	Yes	Yes	Yes	Yes	Yes	No	6
Bachilo	Russia	No	No	No	Yes	Yes	Yes	Yes	Yes	No	5
Bauer	Germany	Yes	No	Yes	Yes	Yes	Yes	Yes	Yes	Yes	8
Bauerle	Germany	Yes	No	Yes	Yes	Yes	Yes	Yes	Yes	Yes	8
Chang	China	Yes	Yes	Yes	Yes	Yes	Yes	Yes	Yes	Yes	9
Chen	China	Yes	No	Yes	Yes	Yes	Yes	Yes	Yes	No	7
Choi	China	Yes	Yes	No	Yes	Yes	Yes	Yes	Yes	Yes	8
Civantos	USA	Yes	Yes	Yes	Yes	Yes	Yes	Yes	Yes	No	8
Consolo	Italy	Yes	Yes	Yes	Yes	Yes	Yes	Yes	Yes	No	8
Fancourt	UK	Yes	Yes	Yes	Yes	Yes	Yes	Yes	Yes	Yes	9
Gao	China	Yes	No	Yes	Yes	Yes	Yes	Yes	Yes	No	7
Guo (Patient)	China	Yes	Yes	Yes	Yes	Yes	Yes	Yes	Yes	No	8
Hu	China	Yes	No	No	No	Yes	Yes	Yes	Yes	No	5
Islam	Bangladesh	No	Yes	Yes	Yes	Yes	Yes	Yes	Yes	Yes	8
Jia	UK	Yes	Yes	Yes	Yes	Yes	Yes	Yes	Yes	No	8
Johnson	Norway	Yes	Yes	Yes	Yes	Yes	Yes	Yes	Yes	No	8
Juanjuan	China	Yes	Yes	Yes	Yes	Yes	Yes	Yes	Yes	No	8
Kantor	USA	Yes	Yes	Yes	Yes	Yes	Yes	Yes	Yes	Yes	9
Khana	India	Yes	No	Yes	Yes	Yes	Yes	Yes	Yes	No	7
Killgore	USA	Yes	Yes	Yes	Yes	Yes	Yes	Yes	Yes	No	8
Lai	China	Yes	Yes	Yes	Yes	Yes	Yes	Yes	Yes	No	8
Lin	China	Yes	Yes	Yes	Yes	Yes	Yes	Yes	Yes	No	8
Liu C	USA	Yes	Yes	No	Yes	Yes	Yes	Yes	Yes	No	7
Liu J	China	Yes	Yes	No	Yes	Yes	Yes	Yes	Yes	No	7
Mahedran	China	Yes	Yes	No	Yes	Yes	Yes	Yes	Yes	Yes	8
Mechili (Students)	Albania	Yes	Yes	Yes	Yes	Yes	Yes	Yes	Yes	Yes	9
Mechili (Family)	Albania	No	No	No	Yes	Yes	Yes	Yes	Yes	No	5
Munoz-Navarro	Spain	Yes	Yes	Yes	Yes	Yes	Yes	Yes	Yes	Yes	9
Naser (General)	Jordan	Yes	Yes	Yes	Yes	Yes	Yes	Yes	Yes	No	8
Naser (Healthcare)	Jordan	Yes	Yes	Yes	Yes	Yes	Yes	Yes	Yes	No	8

Naser (Students)	Jordan	Yes	Yes	Yes	Yes	Yes	Yes	Yes	Yes	No	8
Nguyen	Viet.m	Yes	Yes	Yes	Yes	Yes	Yes	Yes	Yes	No	8
Olaseni	Nigeria	Yes	Yes	No	Yes	Yes	Yes	Yes	Yes	No	7
Pieh	Austria	Yes	No	Yes	Yes	Yes	Yes	Yes	Yes	No	7
Qian (Shangai)	China	Yes	Yes	No	No	Yes	Yes	Yes	Yes	No	6
Qian (Wuhan)	China	Yes	Yes	No	No	Yes	Yes	Yes	Yes	No	6
Que	China	Yes	Yes	Yes	Yes	Yes	Yes	Yes	Yes	No	8
Saddik (General)	UAE	Yes	Yes	Yes	Yes	Yes	Yes	Yes	Yes	No	8
Saddik (Students)	UAE	Yes	Yes	Yes	Yes	Yes	Yes	Yes	Yes	No	8
Salman (Students)	Pakistan	Yes	Yes	Yes	No	Yes	Yes	Yes	Yes	No	7
Salman (Healthcare)	Pakistan	Yes	Yes	Yes	No	Yes	Yes	Yes	Yes	No	7
Sartorao Filho	Brazil	Yes	Yes	No	No	Yes	Yes	Yes	Yes	Yes	7
Shi	China	Yes	Yes	Yes	Yes	Yes	Yes	Yes	Yes	No	8
Sigdel	Nepal	Yes	Yes	No	Yes	Yes	Yes	Yes	Yes	No	7
Solomou	Cyprus	Yes	Yes	Yes	Yes	Yes	Yes	Yes	Yes	No	8
Stickley/Ueda	Japan	Yes	Yes	Yes	Yes	Yes	Yes	Yes	Yes	No	8
Stojanov (Healthcare/COVID)	Serbia	Yes	Yes	No	Yes	Yes	Yes	Yes	Yes	No	7
Stojanov (Healthcare/No-COVID)	Serbia	Yes	Yes	No	Yes	Yes	Yes	Yes	Yes	No	7
Sun	China	Yes	Yes	Yes	Yes	Yes	Yes	Yes	Yes	No	8
Tang	China	Yes	Yes	Yes	Yes	Yes	Yes	Yes	Yes	Yes	9
Temsah	Saudi Arabia	Yes	Yes	Yes	Yes	Yes	Yes	Yes	Yes	Yes	9
Wang	China	Yes	Yes	Yes	No	Yes	Yes	Yes	Yes	No	7
Weilenmann	Switzerland	Yes	No	Yes	Yes	Yes	Yes	Yes	Yes	No	7
Xiao	China	Yes	Yes	Yes	Yes	Yes	Yes	Yes	Yes	Yes	9
Yamamoto	Japan	Yes	Yes	Yes	Yes	Yes	Yes	Yes	Yes	No	8
Zhang (Patient)	China	No	No	No	Yes	Yes	Yes	Yes	Yes	No	5
Zhang (Quarentine)	China	No	No	No	Yes	Yes	Yes	Yes	Yes	No	5
Zhang (General)	China	No	No	No	Yes	Yes	Yes	Yes	Yes	No	5
Zhao M	China	Yes	Yes	Yes	Yes	Yes	Yes	Yes	Yes	Yes	9
Zhao R	China	Yes	No	No	No	Yes	Yes	Yes	Yes	No	5
Zhou	China	Yes	No	Yes	Yes	Yes	Yes	Yes	Yes	No	7
Zhu	China	Yes	Yes	Yes	No	Yes	Yes	Yes	Yes	Yes	8
