# Supplementary material for: Investigating the effect of national government physical distancing measures on depression and anxiety during the COVID-19 pandemic through meta-analysis and meta-regression
Source: Psychol Med. 2021 Mar 2:1–13. doi: 10.1017/S0033291721000933 (PMC7985907; doi:10.1017/S0033291721000933)
Supplement: Supplementary file 1 [file S0033291721000933sup001.zip › S0033291721000933sup001/S0033291721000933sup013.pdf]

Table S3. Mean of social isolation measures implementation national data based on Oxford Covid-19 Government Response Tracker (Hale et al., 2020), during the period of each study.

Author	Country	School closing (2 weeks)	Workplace closing (2 weeks)	Cancel public events (2 weeks)	Restrict. on gather. (2 weeks)	Close public transp. (2 weeks)	Stay-at-home requir. (2 weeks)	Restrict. on internal mov. (2 weeks)	Intern. travel controls (2 weeks)	School closing (4 weeks)	Workplace closing (4 weeks)	Cancel public events (4 weeks)	Restrict. on gather. (4 weeks)	Close public transp. (4 weeks)	Stay-at-home requir. (4 weeks)	Restrict. on internal mov. (4 weeks)	Intern. travel controls (4 weeks)
Ahmad	India	3.000	2.552	2.000	3.448	1.655	2.517	1.793	3.759	2.581	1.721	1.605	2.326	1.116	2.023	1.209	2.907
Ahn	Korea	3.000	2.483	1.621	2.414	0.000	1.172	1.172	3.000	2.997	2.356	1.728	2.591	0.073	1.433	1.396	3.025
Ahorsu (Female)	Iran	2.441	1.814	2.000	0.051	0.712	0.576	1.627	0.000	1.973	1.466	1.671	0.041	0.575	0.466	1.315	0.000
Ahorsu (Male)	Iran	2.441	1.814	2.000	0.051	0.712	0.576	1.627	0.000	1.973	1.466	1.671	0.041	0.575	0.466	1.315	0.000
Alyami	Saudi Arabia	3.000	1.458	1.559	2.220	1.288	1.593	1.729	2.983	2.500	1.506	1.599	1.889	1.210	1.459	1.724	2.479
Amerio	Italy	3.000	3.000	2.000	4.000	0.833	2.438	2.000	3.000	2.565	2.613	1.710	3.419	0.645	2.048	1.774	3.000
Bachilo	Russia	3.000	2.833	2.000	4.000	1.000	3.000	2.000	4.000	3.000	2.768	2.000	3.893	0.893	2.893	2.000	3.893
Bauer	Germany	3.000	2.000	2.000	4.000	0.000	2.000	2.000	4.000	2.894	1.532	2.000	3.362	0.000	1.787	1.681	3.638
Bauerle	Germany	2.662	1.268	1.746	2.746	0.000	1.465	1.366	3.085	2.224	1.059	1.459	2.294	0.000	1.224	1.141	2.576
Chang	China	1.500	1.500	1.444	2.889	1.333	1.000	1.333	0.000	0.844	0.844	0.813	1.625	0.750	0.563	0.750	0.000
Chen	China	1.412	1.412	1.412	2.824	1.294	0.882	1.294	0.000	0.774	0.774	0.774	1.548	0.710	0.484	0.710	0.000
Choi	China	3.000	2.000	2.000	4.000	0.000	1.000	1.000	3.000	3.000	2.184	2.000	4.000	0.053	1.632	1.316	3.000
Civantos	USA	3.000	3.000	2.000	4.000	1.000	2.000	2.000	3.000	3.000	2.850	2.000	3.800	1.000	2.000	1.950	3.000
Consolo	Italy	3.000	3.000	2.000	4.000	1.286	2.600	2.000	3.000	3.000	3.000	2.000	4.000	1.061	2.429	2.000	3.000
Fancourt	UK	2.262	1.646	1.631	3.015	0.708	1.508	1.523	0.000	1.861	1.354	1.342	2.481	0.582	1.241	1.253	0.000
Gao	China	1.263	1.263	1.263	2.526	1.158	0.789	1.158	0.000	0.774	0.774	0.774	1.548	0.710	0.484	0.710	0.000
Guo (Patient)	China	3.000	3.000	2.000	4.000	2.000	2.697	2.000	0.242	2.170	2.170	1.617	3.234	1.574	1.979	1.574	0.170
Hu	China	3.000	3.000	2.000	4.000	2.000	3.000	2.000	1.813	3.000	3.000	2.000	4.000	2.000	3.000	2.000	1.261
Islam	Bangladesh	2.870	2.478	1.696	3.304	1.652	1.087	1.652	2.261	1.784	1.541	1.054	2.054	1.027	0.676	1.027	1.784
Jia	UK	2.786	1.976	1.976	3.714	0.857	1.857	1.881	0.000	2.089	1.554	1.536	2.786	0.643	1.393	1.411	0.000
Johnson	Norway	3.000	2.000	1.364	3.364	1.000	0.000	2.000	4.000	2.250	1.556	0.833	2.389	0.750	0.000	1.278	2.722
Juanjuan	China	3.000	3.000	2.000	4.000	2.000	3.000	2.000	0.000	2.344	2.344	1.813	3.625	1.750	2.063	1.750	0.000
Kantor	USA	3.000	2.294	2.000	3.294	0.882	2.000	1.765	3.000	2.613	1.258	1.645	2.032	0.484	1.097	1.000	2.968
Kha.	India	3.000	3.000	2.000	4.000	2.000	3.000	2.000	4.000	3.000	2.818	2.000	3.879	1.879	2.758	1.939	3.879
Killgore	USA	3.000	3.000	2.000	4.000	1.000	2.000	2.000	3.000	3.000	2.300	2.000	3.400	0.833	1.800	1.700	3.000
Lai	China	1.350	1.350	1.300	2.600	1.200	0.900	1.200	0.000	0.794	0.794	0.765	1.529	0.706	0.529	0.706	0.000
Lin	China	2.636	2.636	2.000	4.000	1.939	2.364	1.939	0.000	1.851	1.851	1.404	2.809	1.362	1.660	1.362	0.000
Liu C	USA	3.000	3.000	2.000	4.000	1.000	2.000	2.000	3.000	3.000	2.862	2.000	3.846	0.985	2.000	1.954	3.000
Liu J	China	3.000	3.000	2.000	4.000	1.778	3.000	2.000	1.556	3.000	3.000	2.000	4.000	1.824	2.824	2.000	1.235
Mahedran	China	1.629	1.629	1.314	2.629	1.257	1.371	1.257	0.000	N.A.	N.A.	N.A.	N.A.	N.A.	N.A.	N.A.	N.A.
Mechili (Students)	Albania	3.000	2.000	2.000	2.000	2.000	2.000	2.000	3.692	2.462	1.538	1.641	1.641	1.436	1.436	1.436	2.897
Mechili (Family)	Albania	3.000	2.000	2.000	2.000	2.000	2.000	2.000	3.692	2.462	1.538	1.641	1.641	1.436	1.436	1.436	2.897
Munoz-.varro	Spain	3.000	2.522	2.000	2.761	0.935	1.870	1.000	3.870	2.400	1.967	1.567	2.133	0.717	1.433	0.800	3.017
Naser (General)	Jordan	2.000	1.571	1.048	2.095	1.048	1.571	1.048	2.667	1.200	0.943	0.629	1.257	0.629	0.943	0.629	1.600
Naser (Healthcare)	Jordan	2.000	1.571	1.048	2.095	1.048	1.571	1.048	2.667	1.200	0.943	0.629	1.257	0.629	0.943	0.629	1.600
Naser (Students)	Jordan	2.000	1.571	1.048	2.095	1.048	1.571	1.048	2.667	1.200	0.943	0.629	1.257	0.629	0.943	0.629	1.600
Nguyen	Viet.m	1.737	1.474	1.316	2.474	0.789	0.947	0.789	2.211	1.269	1.077	0.962	1.808	0.577	0.692	0.577	1.615
Olaseni	Nigeria	3.000	2.292	2.000	4.000	1.000	2.000	1.667	3.000	3.000	2.553	2.000	4.000	1.000	2.000	1.789	3.000
Pieh	Austria	2.000	2.000	1.667	3.333	1.583	1.625	1.583	0.000	1.263	1.263	1.053	2.105	1.000	1.026	1.000	0.000
Qian (Shangai)	China	3.000	3.000	2.000	4.000	2.000	3.000	2.000	0.000	2.417	2.417	1.833	3.667	1.778	2.167	1.778	0.000
Qian (Wuhan)	China	3.000	3.000	2.000	4.000	2.000	3.000	2.000	0.000	2.417	2.417	1.833	3.667	1.778	2.167	1.778	0.000
Que	China	3.000	3.000	2.000	4.000	1.000	2.720	2.000	3.000	3.000	3.000	2.000	4.000	0.692	2.462	2.000	3.000
Saddik (General)	UAE	3.000	1.800	1.877	3.077	0.985	1.800	1.400	3.800	2.797	1.620	1.544	2.532	0.810	1.481	1.152	3.658

Saddik (Students)	UAE	2.240	0.840	0.480	0.000	0.000	0.360	0.000	3.000	1.436	0.538	0.308	0.000	0.000	0.231	0.000	2.385
Salman (Students)	Pakistan	3.000	2.173	2.000	4.000	1.867	1.867	1.813	3.813	3.000	1.831	1.798	3.596	1.573	1.573	1.528	3.685
Salman (Healthcare)	Pakistan	3.000	2.280	2.000	4.000	2.000	2.000	1.960	4.000	3.000	2.203	2.000	4.000	1.844	1.844	1.781	3.953
Sartorao Filho	Brazil	3.000	3.000	2.000	4.000	2.000	1.962	2.000	4.000	3.000	3.000	2.000	3.850	2.000	1.625	2.000	4.000
Shi	China	3.000	3.000	2.000	4.000	2.000	3.000	2.000	1.280	3.000	3.000	2.000	4.000	2.000	2.951	2.000	0.780
Sigdel	Nepal	3.000	3.000	2.000	4.000	1.920	2.000	2.000	4.000	2.366	1.950	1.592	2.876	0.945	1.539	1.428	2.083
Solomou	Cyprus	3.000	2.000	2.000	3.429	1.619	1.619	1.619	4.000	2.400	1.429	1.771	2.057	0.971	0.971	0.971	3.086
Stickley/Ueda	Japan	3.000	1.000	1.000	0.000	0.000	0.632	1.000	2.842	3.000	1.000	1.000	0.000	0.000	0.387	1.000	2.516
Stojanov (Healthcare/COVID)	Serbia	3.000	3.000	2.000	4.000	2.000	3.000	2.000	4.000	3.000	3.000	2.000	4.000	2.000	3.000	2.000	4.000
Stojanov (Healthcare/No-COVID)	Serbia	3.000	3.000	2.000	4.000	2.000	3.000	2.000	4.000	3.000	3.000	2.000	4.000	2.000	3.000	2.000	4.000
Sun	China	3.000	2.778	2.000	4.000	1.222	2.833	1.917	2.444	3.000	2.840	2.000	4.000	1.440	2.880	1.940	2.160
Tang W	China	3.000	3.000	2.000	4.000	2.000	3.000	2.000	0.273	2.750	2.750	2.000	4.000	2.000	2.500	2.000	0.167
Temsah	Saudi Arabia	0.000	0.000	0.000	0.000	0.000	0.000	0.000	0.000	0.000	0.000	0.000	0.000	0.000	0.000	0.000	0.000
Wang	China	3.000	3.000	2.000	4.000	2.000	3.000	2.000	0.727	3.000	3.000	2.000	4.000	2.000	2.778	2.000	0.444
Weilenmann	Switzerland	3.000	2.591	2.000	3.591	0.000	0.864	0.864	3.000	1.917	1.583	2.000	2.583	0.000	0.528	0.528	1.917
Xiao	China	2.348	2.348	1.913	3.826	1.826	1.957	1.826	0.000	1.459	1.459	1.189	2.378	1.135	1.216	1.135	0.000
Yamamoto	Japan	3.000	1.000	1.000	0.000	0.000	1.000	1.000	3.000	3.000	1.000	1.000	0.000	0.000	1.000	1.000	3.000
Zhang (Patient)	China	3.000	3.000	2.000	4.000	2.000	3.000	2.000	0.000	2.273	2.273	1.758	3.515	1.697	2.000	1.697	0.000
Zhang (Quarentine)	China	3.000	3.000	2.000	4.000	2.000	3.000	2.000	0.000	2.273	2.273	1.758	3.515	1.697	2.000	1.697	0.000
Zhang (General)	China	3.000	3.000	2.000	4.000	2.000	3.000	2.000	0.000	2.273	2.273	1.758	3.515	1.697	2.000	1.697	0.000
Zhao M	China	2.182	2.182	1.818	3.636	1.727	1.773	1.727	0.000	1.333	1.333	1.111	2.222	1.056	1.083	1.056	0.000
Zhao R	China	3.000	3.000	2.000	4.000	2.000	2.500	2.000	0.000	1.853	1.853	1.471	2.941	1.412	1.588	1.412	0.000
Zhou	China	3.000	3.000	2.000	4.000	2.000	3.000	2.000	1.818	3.000	3.000	2.000	4.000	2.000	3.000	2.000	1.111
Zhu Z	China	2.824	2.824	2.000	4.000	2.000	2.176	2.000	0.000	1.548	1.548	1.290	2.581	1.226	1.258	1.226	0.000
